# Node of origin matters: comparative analysis of soil water limitation effects on nodal root anatomy in maize (*Zea mays*)

**DOI:** 10.1093/aob/mcaf075

**Published:** 2025-05-15

**Authors:** Tina Koehler, Yunhee Kim, Shu-Yin Tung, Adrien Heymans, Nicolas Tyborski, Franziska Steiner, Andreas J Wild, Johanna Pausch, Mutez A Ahmed, Hannah M Schneider

**Affiliations:** Root–Soil Interaction, TUM School of Life Sciences, Technical University of Munich, Munich, Germany; Root–Soil Interaction, TUM School of Life Sciences, Technical University of Munich, Munich, Germany; Institute for Agroecology and Organic Farming, Bavarian State Research Center for Agriculture (LfL), Freising, Germany; TUM School of Life Sciences, Technical University of Munich, Munich, Germany; Umeå Plant Science Centre, Swedish University of Agricultural Sciences, Umeå, Sweden; Ecological Microbiology, Bayreuth Center of Ecology and Environmental Research (BayCEER), University of Bayreuth, Bayreuth, Germany; Soil Science, TUM School of Life Sciences, Technical University of Munich, Munich, Germany; Agroecology, Bayreuth Center of Ecology and Environmental Research (BayCEER), University of Bayreuth, Bayreuth, Germany; Agroecology, Bayreuth Center of Ecology and Environmental Research (BayCEER), University of Bayreuth, Bayreuth, Germany; Root–Soil Interaction, TUM School of Life Sciences, Technical University of Munich, Munich, Germany; Genetics and Physiology of Root Development, Leibniz Institute for Plant Genetics and Crop Plant Research (IPK), Gatersleben, Germany; Division of Crop Plant Genetics, Department of Crop Science, Georg-August-University Goettingen, Goettingen, Germany

**Keywords:** Drought, root anatomy, shoot node, adventitious root, maize, *Zea mays* L, field plot experiment

## Abstract

**Background and Aim:**

Root anatomy, determining the composition and organization of root tissues, has implications for water uptake and transport, and potential for enhancing crop resilience amid changing environmental conditions and erratic water supply. While our understanding of the functional relationship between root anatomical traits and soil resource acquisition continues to improve, anatomical traits are commonly investigated on adventitious roots emerging from a single node or averaged across nodes. We test the hypothesis that drought adaptations of anatomical and hydraulic phenes are specific to the nodal origin of the root.

**Methods:**

We grew four maize (*Zea mays* L.) genotypes in the field under control and drought conditions, imposed by rainout shelters. Subsequently, we investigated the effect of soil drought on crown root anatomical phenes between consecutive shoot nodes. Based on these phenotypes, we inferred root cross-sectional hydraulic properties by integrating simulations of root anatomical networks via the GRANAR model and translating the outputs into hydraulic properties using the MECHA model.L.

**Key Results:**

At the individual node level, drought-induced changes in root anatomical and hydraulic phenes were neither consistently significant nor unidirectional across nodes or genotypes. Notably, only second node crown roots consistently exhibited significant changes in response to drought. However, we observed distinct treatment differences in the development of phenes between consecutive shoot nodes. Most root anatomical and hydraulic phenes showed a (hyper)allometric relationship with increasing root cross-sectional area from older to younger roots. However, under drought, those allometric trajectories shifted. Specifically, root cross-sectional area and the areas of stele, cortex, metaxylem and aerenchyma, as well as cortical cell size and the axial hydraulic conductance increased more strongly from older to younger roots under drought. In contrast, metaxylem number increased more strongly under controlled conditions.

**Conclusion:**

Our findings suggest that examining the drought response of root anatomical phenes at a single node may not provide a comprehensive understanding of root system responses to the environment.

## INTRODUCTION

Global warming results in amplified atmospheric drying (i.e. increasing vapour pressure deficit), which increases evapotranspiration (Will *et al.*, 2013; Novick *et al.*, 2024) and accelerates soil drying (Zhou *et al.*, 2019). The mismatch between the co-occurring increase in atmospheric water demand and the decrease in soil water supply may regionally lead to plant water deficit (Tardieu *et al.*, 2018). When lasting for extended periods or occurring during phases critical for yield determination, water deficit becomes an increasing threat to humanity, as it leads to reduced growth rates, translating into lower grain yields and resulting in food security issues (Lobell and Gourdji, 2012; López *et al.*, 2021).

Conventional breeding for high productivity involves selecting plants based on their yield performance under optimal, high-input conditions. Slowly, awareness is rising that this might not be enough to face the increasing frequency and intensity of erratic climate conditions, considering that yields of many crop species have been shown to stagnate in recent years (Ray *et al.*, 2012). Modern breeding strategies increasingly consider performance under a range of conditions, including suboptimal or stress-prone environments. However, they mainly focus on aboveground performance with little consideration of root systems. Roots are at the forefront of managing crop resource acquisition (Lynch, 2022). While the architecture of the root system primarily governs spatiotemporal acquisition of soil resources (Lynch, 1995; Klein *et al.*, 2020), anatomical phenes (i.e. characteristics of the composition and organization of root tissues at a cellular level that contribute to its phenotype like genes contribute to a genotype) are associated with the water channelling ability of the plant (Klein *et al.*, 2020), making root anatomical traits a possible target for improving yield and drought resilience (Lynch, 2007; Lynch and Brown, 2012; Bauw *et al.*, 2019; Schneider *et al.*, 2023).

Multiple root anatomical phenes have been proposed to plastically adapt to environmental conditions (Schneider and Lynch, 2020), such as water availability and soil physicochemical properties, with this plasticity being genetically controlled to some extent (Schneider *et al.*, 2020). Such adaptations can potentially enhance crop performance under drought through (1) enabling soil water conservation via changes in root hydraulic conductance, (2) reducing metabolic costs of root maintenance and (3) increasing tensile strength to enable penetration of dry, compacted soil (Lynch *et al.*, 2014, 2021; Klein *et al.*, 2020). For example, root metaxylem size and abundance are related to hydraulic safety (Martínez-Vilalta *et al.*, 2002; Li *et al.*, 2009) and the water channelling ability from the root to the shoot (axially; e.g. Richards and Passioura, 1989; Souza *et al.*, 2013; York *et al.*, 2015; Affortit *et al.*, 2024b). According to Hagen–Poiseuille’s law, a reduction in vessel radius reduces the vertical water transport capacity from the root to the shoot (i.e. axial conductance, K_x_), potentially enabling water saving if the axial conductance were to limit the overall root conductance, especially in deeper soil layers. Less obviously, a reduction in the number of metaxylem vessels is also linked to a decrease in root radial hydraulic conductivity (*k*_r_), impairing the root’s efficiency in water uptake (Heymans *et al.*, 2020). This effect is due to the increased distance between the soil–root interface and the xylem vessels, lengthening the apoplastic and symplastic pathways. For the same reason, a thicker cortex would reduce *k*_r_ (Rieger and Litvin, 1999). Likewise, an increase in the proportion of root cortical aerenchyma, i.e. air-filled spaces that develop in the root cortex due to programmed cell death (Drew *et al.*, 2000; Klein *et al.*, 2020), leads to a decrease in *k*_r_ due to longer path lengths in the apoplastic and symplastic pathways (Fan *et al.*, 2007). In contrast, a relative increase in stele area is linked to higher *k*_r_ because it shortens the apoplastic pathway by ‘bringing the xylem vessels closer’ to the interface with the soil (Heymans *et al.*, 2020). Additionally, roots can develop apoplastic barriers by developing Casparian bands (i.e. the suberization and lignification of the exodermal and/or endodermal cell walls; Enstone *et al.*, 2002). While this blockage of the apoplastic pathway increases the resistance to radial water flow and uptake (Zimmermann and Steudle, 1998), it also restricts radial water loss from the root to the soil (Ranathunge *et al.*, 2011). This process of hydraulic redistribution happens when soil water is distributed heterogeneously across the root system (Neumann *et al.*, 2014).

Root anatomy is also a key factor affecting the metabolic investment in root formation and maintenance, as some tissues demand more metabolic resources than others (Lynch *et al.*, 2021). For instance, the metabolic cost of sustaining the root cortex can be lowered by replacing the cortical tissue with air through the formation of root cortical aerenchyma (White and Hammond, 2008; Zhu *et al.*, 2010; Chimungu *et al.*, 2015*b*; Klein *et al.*, 2020). Likewise, larger cortical cells arranged in fewer cell files may lower the cortical metabolic demand (Jaramillo *et al.*, 2013; Chimungu *et al.*, 2014*a*, *b*; Klein *et al.*, 2020; Lopez-Valdivia *et al.*, 2024; Sidhu and Lynch, 2024). Reducing the metabolic cost of the maintenance of any tissue potentially benefits the plant by increasing the availability of internal resources for processes such as root growth (enhancing soil exploration) or the growth of photosynthetic tissues, ultimately improving plant productivity and overall growth (Lynch *et al.*, 2021).

As the soil dries, soil strength and mechanical impedance to root growth increase non-linearly (Bengough *et al.*, 2011), potentially imposing physical limitations on root growth. Root anatomical traits that enhance tensile strength can facilitate continued access to deep soil resources (Chimungu *et al.*, 2015*a*). For example, root and especially stele radius are positively related to soil penetrability, enhancing the ability of roots to overcome the high mechanical impedance of desiccated, hardened soils (Materechera *et al.*, 1991; Clark *et al.*, 2008; Meijer *et al.*, 2024). Similarly, smaller cortical cells in maize were demonstrated to enhance root tensile strength (Chimungu *et al.*, 201*5*a) and growth in desiccated, compacted soils (Klein *et al.*, 2020). In addition, multiseriate cortical sclerenchyma, i.e. outer cortical small cells with thick lignified walls, was shown to improve the mechanical strength of the root to support continued elongation in compacted soils (Schneider, 2022).

While the effect of one phene (e.g. cortex area) on a single function (i.e. water uptake and transport, metabolic cost, or soil penetrability) might be physically or physiologically predictable, it can create trade-offs with another phene or another function. For example, roots with a small number of cortical cell layers and large cortical cells are suggested to have a reduced metabolic cost (Lynch *et al.*, 2014). However, few cortical layers consisting of large cortical cells result in a high radial root conductivity and, hence, potentially excessive water use (Heymans *et al.*, 2020). Similarly, a high proportion of root cortical aerenchyma might reduce the root’s metabolic burden but also decreases the root’s tensile strength and *k*_r_. A large stele area may contribute to enhanced soil penetrability but leads to a high radial hydraulic conductivity, lowering the water-saving potential. Experimentally quantifying the effects of several interacting phenes on specific functions is technically challenging. As a result, most discussions are limited to qualitative assessments, where the varying importance of different processes cannot be quantitatively accounted for, and implications remain elusive. Klein *et al.* (2020) have recently highlighted the need to consider phene aggregates, or the integrated phenotype, for explaining root-related performance differences under stress.

In light of these complexities, incorporating an additional layer of consideration may seem daunting, but it is crucial for a comprehensive understanding and optimization of root phenes in relation to water use. The maize root system initially comprises the primary root emerging from the radicle and the seminal roots emerging from the scutellar node. Successively, whorls of nodal roots emerge from shoot nodes. Collectively, these shoot-borne roots are called adventitious roots, and more specifically crown roots when they emerge from belowground stem nodes (Hochholdinger, 2009). Several studies have evaluated root anatomical phenotypes of the second or fourth shoot node (Burton *et al.*, 2013; Saengwilai *et al.*, 2014; Schneider *et al.*, 2020) or averaged across all nodes (Jordan *et al.*, 1993; York and Lynch, 2015). However, to our knowledge, how root anatomical phenes vary by nodal position has never been characterized under conditions of soil-water limitations. Yang *et al.* (2019) have recently highlighted the variability of root anatomical responses to nitrogen limitation across roots from consecutive node orders. Given that the two most common forms of nitrogen in soil, ammonium and nitrate, are dissolved in soil water and hence primarily transported to plants through water flow (Ding *et al.*, 2018), we hypothesize that root anatomical changes in response to water stress are likewise specific to the node order from which roots emerge.

Here, we focus on two key objectives. First, we aim to identify which root anatomical phenes are most responsive to soil drought under field conditions while accounting for variation across nodal positions and genotypes (maize landraces and modern genotypes). Recognizing the challenge of translating specific phenes into individual functions and individual functions into whole-root responses, our second objective is to evaluate how anatomical adaptations influence root cross-section hydraulic properties in response to drought. By parameterizing a digital root anatomical network and integrating it with a hydraulic model, we estimate the root cross-sectional hydraulic properties and explore their role in drought adaptation.

## MATERIALS AND METHODS

### Plant material and field conditions

This study was conducted as an experiment under field conditions in 2022 in Schönburg, Pocking, Germany (lat. 48.382261, long. 13.263678). From a total of 12 maize genotypes grown, we investigated the root anatomy of four genotypes in detail: two landraces (i.e. year of release pre-1945) and two modern genotypes (Table 1). The genotypes were chosen based on contrasting water use responses and performance under drought in a preceding glasshouse experiment (Koehler *et al.*, 2023*a*; Steiner *et al.*, 2024; Tyborski *et al.*, 2024; Wild *et al.*, 2024). Briefly, genotypes GB and SE exhibited a relatively low water use efficiency and a marked decrease in biomass due to soil drought, while SC and WE demonstrated a higher water use efficiency and a relatively smaller reduction in biomass during soil drought (Wild *et al.*, 2024). Further, GB displayed greater stomatal sensitivity to soil drying (i.e. initiation of stomatal closure in already relatively wet soil conditions) compared to SE, SC and WE (Koehler *et al.*, 2023a).

**Table 1.** Characteristics of the genotypes investigated.

Abbreviation	Variety	Landrace/modern	Type	Maturity	Origin	Year of release
SC	Braunes Schindelmeiser	Landrace	Grain	Early	Germany	before 1945
GB	Gelber Bad. Landmais	Landrace	Silage	Medium	Germany	before 1945
SE	PM Serveza	Modern	Grain	Early	Germany	2018
WE	Weihenstephaner 2	Modern	Grain	Late	Germany	2016

The experiment was arranged in a complete randomized block design with four blocks (Tyborski *et al.*, 2024). Each genotype was grown in four rainfed replicate plots (3 × 4 m), one per block (Supplementary Data Fig. S1). One plot consisted of four rows of plants of the same genotype (distance between rows: 70 cm, planting density: 9–10 plants m^−2^). Plants were sown on 21 April 2022 and grown under conventional agricultural practices with 135 kg N ha^−1^ organic nitrogen fertilizer (residual sludge) applied before sowing. Additionally, 57 kg N ha^−1^ mineral nitrogen fertilizer (Alzon, SKW, Lutherstadt Wittenberg, Germany) and 3 L herbicide ha^−1^ (2 L Laudis ha^−1^ and 1 L Spectrum ha^−1^) were applied 24 d after sowing (DAS).

To generate a water-reduced treatment, we installed UV-transparent rain-out shelters above four plots per genotype 26–30 DAS. The rain-out shelters covered 60 % of the area underneath to achieve an equivalent reduction in precipitation (Tyborski *et al.*, 2024). The drought treatment did not significantly affect the microclimate at the canopy level (Supplementary Data Fig. S2) which was measured by two temperature and relative humidity loggers per drought treatment (EL-USB-2, LASCAR electronics, Whiteparish, UK).

The loess-derived soil classifies as Haplic Luvisol with a slightly silty clay texture in the topsoil (IUSS Working Group WRB, 2022; Tyborski *et al.*, 2024). Soil water content dynamics were monitored periodically (once a week) by Pino-Tech SoilWatch 10-sensors that were installed at 30 and 60 cm depth in each plot (UAB Eltechnika, Vilnius, Lithuania). The SoilWatch 10-sensors were calibrated using *in situ* soil cores collected from each depth. These cores were first saturated with water and then dried at room temperature, while soil water content was continuously recorded using TEROS10 water content sensors (METER Environment, Munich, Germany) to infer water content from SoilWatch 10-sensor output voltage. Soil water potential dynamics were monitored hourly at 30 cm depth by TEROS21-sensors installed in three out of four replicate plots per treatment (METER Environment).

### Harvest and root sampling

From 110 to 115 DAS, when the plants were at the ripening stage (BBCH 85, BBCH-scale from Biologische Bundesanstalt, Bundessortenamt und Chemische Industrie), we sampled above- and belowground biomass from three randomly selected plants per plot to assess the root:shoot ratio. During this sampling campaign, we also assessed the specific rhizosheath mass, i.e. rhizosheath weight per root area (with root area approximated by root weight). Note that although root biomass dry weight was shown to be a reasonable indicator of root length (*R*^2^ = 0.8, and hence root surface area) in a preceding glasshouse experiment (Koehler *et al.*, 2023*a*) that investigated the same genotypes as in this study, root biomass dry weight does not account for root anatomical adaptations to drought, such as the formation of aerenchyma or the development of thicker roots. However, it was considered functional in the present study to account for differential amounts of collected roots from which rhizosheath was extracted (Steiner *et al.*, 2024).

Rhizosheath, i.e. soil attached to the root after excavation (McCully, 1999), serves as an integrated indicator of rhizosphere processes such as root exudation, bacterial exopolysaccharide (EPS) production, root hair formation, mycorrhizal fungi association and root architectural properties (Cheraghi *et al.*, 2023). Such rhizosphere processes may be seen as an extension of the root radius effective in water uptake (Carminati *et al.*, 2017). We manually excavated three plants per plot from the inner two rows by removing a soil block of 40 × 20 × 25 cm, with the plant base positioned approximately at the centre of the block. Roots with adhering soil were collected for a defined time of 12 min and carefully shaken by hand. The soil that remained attached to the roots after shaking was defined as the rhizosheath. It was detached from the roots by immersed wet sieving. Rhizosheath mass was determined after drying at 60 °C. Root and shoot biomass were dried at 105 °C for 24 h and weighed.

**Fig. 1.**
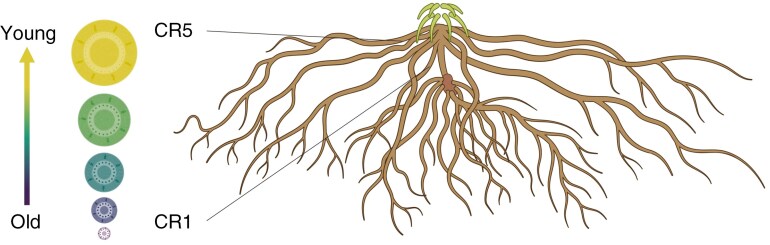
Roots were sampled from consecutive nodal positions representing a temporal gradient of root development under progressive soil drying, ranging from crown roots at node 1 (CR1) to those at node 5 (CR5). Root anatomical properties were analysed, forming the basis for constructing virtual root anatomical networks (exemplary cross-sections represent roots from genotype SC), which were then used to estimate root cross-sectional hydraulic properties. Root system visualized with BioRender.com.

From 151 to 158 DAS, after the plants had reached physiological maturity (BBCH 94), we sampled aboveground biomass to assess plant performance in terms of shoot biomass and grain yield. We also collected roots from different nodal positions for anatomical analysis during this sampling campaign. Half of the root system was excavated for three randomly selected plants per plot by removing a soil block (29 × 18 × 9 cm), centred horizontally at the plant base. The excavated root crowns were first soaked and subsequently washed in tap water. From the clean roots, we determined the number of crown roots per node, excised every node and preserved one representative root per node in 75 % (v/v) ethanol as sampled 10 cm from the root base, resulting in the following root samples (Fig. 1): crown roots of node 5 (CR5 – youngest/most recently developed roots), crown roots of node 4 (CR4), crown roots of node 3 (CR3), crown roots of node 2 (CR2) and crown roots of node 1 (CR1 – oldest roots). Roots of the same genotype × drought treatment × node were pooled from the replicate plots. Aboveground biomass of five random plants per plot was separated into shoot components (leaf, stem, husk, undeveloped ears), shelled grains and cob. The separate parts were pooled by plot, dried at 105 °C for 24 h and weighed.

### Image analysis

The image generation and processing were done at the Centre for Crop Systems Analysis, Wageningen University (WUR), Wageningen, Netherlands. From pooled roots of each node, drought treatment and genotype, transverse sections with comparable distance to the plant base were hand-sectioned using a razor blade and imaged under a light microscope (Kern Optics OBF-1) with a camera (Kern Optics ODC 832) at 4× and 10× magnification. Images were analysed using ImageJ v.1.54e (Rasband 1997–2018), in combination with the ObjectJ plugin, in which cortex, stele, aerenchyma and xylem vessels were manually outlined, and cell files manually counted as described in detail in Yang *et al.* (2019). We quantified the following parameters: root cross-sectional area (RXA), stele area (SXA), cortex area (CXA), cortex:stele ratio (CS), cortical cell size (CCS), cortical file number (CFN), metaxylem number (MXN), total metaxylem area (MXA), aerenchyma area (AXA) and aerenchyma per cent (AXAP; Supplementary Data Fig. S3).

### Estimation of hydraulic properties emerging from root anatomical features

To estimate how root anatomical features collectively translate into root hydraulic properties, we parameterized the Generator of Root Anatomy in R (GRANAR v.1.1; https://granar.github.io, Heymans *et al.*, 2020) and fed the outcome into the Model of Explicit Cross section Hydraulic Architecture (MECHA, v.2.1; https://mecharoot.github.io, Couvreur *et al.*, 2018). GRANAR generates digital root anatomies on the basis of the measured anatomical parameters (Fig. 1). Subsequently, MECHA uses the digital root anatomies to infer hydraulic properties, i.e. root radial hydraulic conductivity (*k*_r_, for the water pathway ‘permeability’ from the soil–root interface to xylem vessels), radial hydraulic conductance (*K*_r_, for the effective radial water transport capacity) and axial hydraulic conductance (*K*_x_, for the effective axial water transport capacity). The subcellular hydraulic parameters are the same as in Heymans *et al.* (2020), and the chosen hydraulic scenario accounts for the hydrophobic structures of an endodermal Casparian strip, which we assumed were developed in the root segments collected due to their position close to the root base. The script used is an updated version of the workflow used in McLaughlin *et al.* (2024), available on GitHub (RootDiversity v.1.2.1; doi: 10.5281/zenodo.14045758).

### Data and statistical analysis

Data processing, statistical analyses and visualizations were generated using R v.4.3.2 (R Core Team, 2023). PERMANOVA was conducted with PRIMER v.7.0.23 (PRIMER-e, Auckland, New Zealand).

### Soil water dynamics

To assess differences in soil moisture dynamics, the area under the curve (AUC) of soil water content over time was computed for each plot at both depths (Supplementary Data Figs S4 and S5). The statistical analysis involved the following steps. First, the normality of the AUC data was confirmed to ensure the validity of subsequent analyses. Subsequently, we initially fitted a linear mixed effect model including block as a random factor and drought treatment and genotype as fixed factors, as well as their interaction [lmer() function in R, lme4 package; Bates *et al.*, 2015]. However, this model faced convergence issues, indicating overparameterization due to the minimal contribution of block effects. Consequently, we simplified the model by removing the random effect, fitting a linear model including the factors drought treatment (T) and genotype (G), as well as their interaction term [T:G, lm() function in R, stats package, R Core Team, 2023]. Subsequently, we confirmed the validity of our analysis by verifying that the pre-conditions for the linear model were met. This involved examining diagnostic plots to assess the model assumptions, including normality of residuals, homoscedasticity and linearity. Lastly, we tested if the relative response to the drought treatment differed between genotypes overall by applying an ANOVA on the model [anova() function in R, stats package; R Core Team, 2023].

Soil water content was used as the primary indicator of water stress (Supplementary Data Figs S4 and S5) as soil water potential data are not consistently available at the plot level throughout the experimental period due to occasional sensor malfunctions or instances where the soil water potential fell outside the measurable range. Available time series data of soil water potentials can be found in Fig. S6.

### Plant performance

Drought treatment differences and genotypic differences in plant performance (i.e. shoot vegetative dry biomass and grain yield at 14 % seed moisture content) were tested according to the same procedure described above. To meet the assumption of normality, we applied a log-transformation to the response variables (shoot vegetative dry biomass and grain yield) before the analysis. We first fitted a linear mixed-effects model with block as a random factor and drought treatment, genotype and their interaction as fixed factors. This linear mixed effect model likewise suggested that block as a random factor does not contribute to explaining more variance in the data. Consequently, we simplified the analysis by fitting a linear model including the factors of drought treatment (T) and genotype (G), as well as their interaction (T:G). The following analysis was conducted as specified above. The same analysis was applied for ‘belowground performance’ (i.e. root biomass, root:shoot ratio and specific rhizosheath mass; Supplementary Data Fig. S7).

### Phene-independent integrated root anatomy

To understand the drought treatment effect on phene-independent integrated root anatomy, we conducted a multivariate root anatomy analysis, considering all phenes that collectively define root anatomy. For that, we used permutational multivariate analysis of variance (PERMANOVA, Anderson, 2001), considering the five root nodal positions (CR1–CR5), landrace vs. modern cultivars and the drought treatments (drought vs. control) as fixed factors, and genotype nested in landrace (SC, GB) vs. modern cultivar (SE, WE) as a random factor. Note that block could not be accounted for as a random factor due to the pooling of samples during sampling. First, we calculated Euclidean distances on log(*x* + 1)- and *z*-transformed values of all measurements. In the following, we used PERMANOVA with sums of squares type III and permutation of residuals under a reduced model (Anderson, 2001) in PRIMER v.7.0.23 (PRIMER-e). Considering the insignificance of landraces vs. modern cultivars and any related interaction (Supplementary Data Table S1), this categorization was excluded from further analysis. The subsequent pairwise comparison focused on the five nodal positions (R: CR1–CR5) and the drought treatments (T: drought vs. control) as fixed factors, with genotype (G: SC, GB, SE, WE) treated as a random factor.

### Root anatomical phenes per nodal position

We examined variations in individual root anatomical phenes across nodal positions, drought treatments and genotypes according to the same procedure as described for plant performance with the difference of directly applying a linear model as, due to the pooling of samples, block could not be accounted for as a random factor. Given that a significant three-way interaction among root nodal position, drought treatment and genotype was consistently observed at the phene level, we explored differences between drought treatments within each level of the factors genotype and nodal position by calculating the estimated marginal means [emmeans() function and package in R; Lenth, 2024]. Pairwise comparisons were then conducted on these estimated marginal means [contrast() function in R in the stats package with the interaction set to ‘pairwise’ and grouped by genotype and root nodal position]. The results were visualized as the mean relative difference between treatments (see Fig. 6).

### Root anatomical phenes integrated over the whole root system

**Fig. 2.**
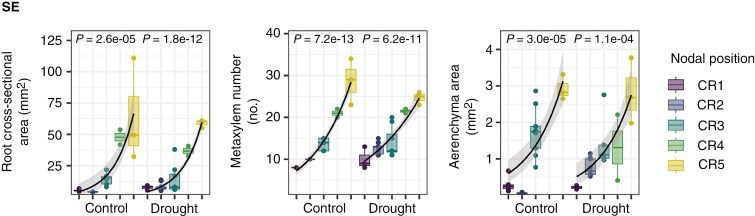
Expression of exemplary phenes dependent on nodal position, i.e. age (from the oldest root – CR1 to the youngest root – CR5) per genotype (genotype SE presented here) and drought treatment. The black line represents the fit of a generalized linear model (GLM, with a log link function, allowing for exponential relationships). The *P*-value of ≤0.05 indicates that the GLM describes the data significantly. From the GLM, the slope was extracted to characterize the change of an anatomical phene across nodes (i.e. with age) between treatments. Plots for all root anatomical phenes and genotypes can be found in Supplementary Data Fig. S7.

To get an integrated understanding of changes in root anatomical phenes between drought treatments per genotype across nodal origin, we analysed the change of a respective phene with relative root age (i.e. from the oldest root CR1 to the youngest root CR5) per genotype and treatment. To do that, we fitted a generalized linear model [GLM, with a log link function, allowing for exponential relationships, glm() function in the stats package] between nodal positions (translated into equally spaced relative root age per node) and the expression of each anatomical phene per genotype with an interaction term for the drought treatment. If the *P*-value of the slope was significant for the reference level (≤0.05), we concluded that the expression of a particular root anatomical phene exhibited a statistically significant directed change over time (i.e. through the development of roots from subsequent nodes, Fig. 2). If the slope-interaction term with the drought treatment was significant, we concluded that the change in the expression of a particular root anatomical phene over time differed significantly between the two drought treatments. We extracted and compared the resulting slope ± standard error, which is a measure of change in a phene across nodes, i.e. with age (from CR1 to CR5) between drought treatments per genotype as an integrative drought response indicator.

### Root anatomy response to drought treatment

Lastly, we related the dynamics of water content time series (AUC) to the above-mentioned root anatomy drought response indicator (Fig. 2) across the root system in a simple linear model [lm() function in R] to see whether there was a directed adaptation in root phenes to soil drought over time. When the change in a certain phene with age was non-significant (Supplementary Data Fig. S8), we considered this as the baseline development (in the case of the control treatment) or potential adaptation strategy (in the case of the drought treatment) for a given genotype × treatment combination and included it in the analysis.

## RESULTS

### Soil water dynamics differ between drought treatments

**Fig. 3.**
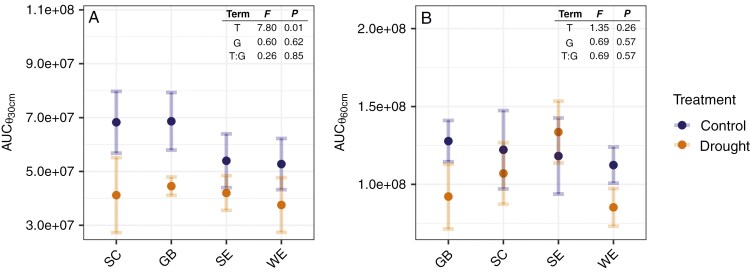
Area under the curve (AUC) of soil water content at 30 cm soil depth (A, θ_30cm_), and soil water content at 60 cm soil depth (B, θ_60cm_) over time per genotype (*x*-axis) between drought treatments. AUC serves to quantify the integration of absolute dryness and the temporal dynamics of soil moisture depletion. The whole time series of θ_30cm_ and θ_60cm_ can be found in Supplementary Data Figs S4 and S5. Effects of drought treatment (T), genotype (G) and their interaction (T:G) on soil water content dynamics based on a linear model are indicated in the upper right corner.

The rainout shelter treatment significantly affected soil moisture dynamics (Supplementary Data Figs S3 and S4) and absolute dryness (Fig. 3). The average area under the curve of soil water content over time at 30 cm depth, AUC (θ_30cm_), was significantly smaller in the drought treatment (Fig. 3A, Term = T). Soil in the drought treatment was 22–40 % drier than in the control treatment at 30 cm soil depth. At 60 cm depth, the difference between drought treatments was not significant. The average area under the curve of soil water content over time at 60 cm depth, AUC (θ_60cm_), tended to be lower in the drought treatment, but the difference was not statistically significant (Fig. 3B, Term = T), suggesting that water availability was comparable between drought treatments at depth. Genotypic differences in soil moisture dynamics were not statistically significant at either depth (Term = G). Likewise, relative differences in soil moisture dynamics between drought treatments (Term = T:G) did not vary significantly between genotypes.

### Plant performance decreased due to drought

**Fig. 4.**
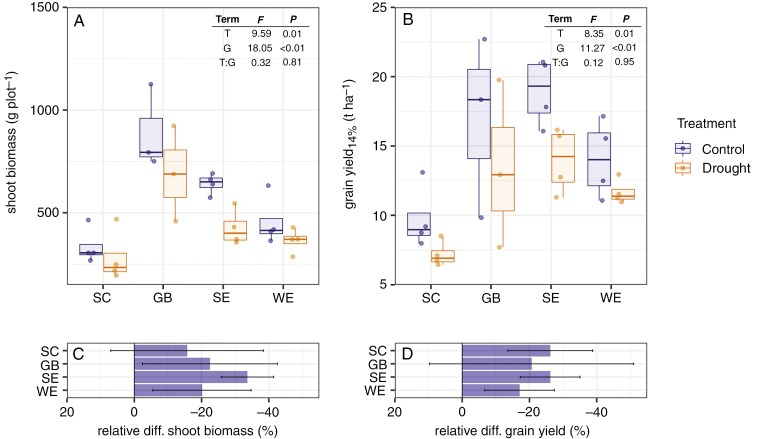
Plant performance between drought treatments in terms of shoot vegetative dry biomass (A) and grain yield (B). Effects of drought treatment (T), genotype (G) and their interaction (T:G) on plant performance based on a linear model are indicated in the upper right corner. Plant performance between genotypes in terms of relative difference in shoot vegetative dry biomass (C) and in grain yield at 14 % moisture content (D) between control and non-drought treatments.

Plants responded significantly to soil drought induced by the rainout shelters with a 16–34 % decrease in vegetative biomass (Fig. 4A) and a 17–26 % decrease in grain yield (Fig. 4B, Term = T), on average. Plant performance differed significantly between genotypes (Term = G). However, relative differences in vegetative biomass (Fig. 4A, Term = T:G, Fig. 4C) and grain yield (Fig. 4B, Term = T:G, Fig. 4D) between drought treatments did not vary between genotypes.

Plants significantly increased root biomass and root:shoot ratio under drought conditions with insignificant genotypic differences in the relative response to soil drought (Supplementary Data Fig. S7A–B). Specific rhizosheath mass did neither differ between drought treatments nor between genotypes (Fig. S7C).

### Root anatomical–hydraulic properties under soil drought

**Fig. 5.**
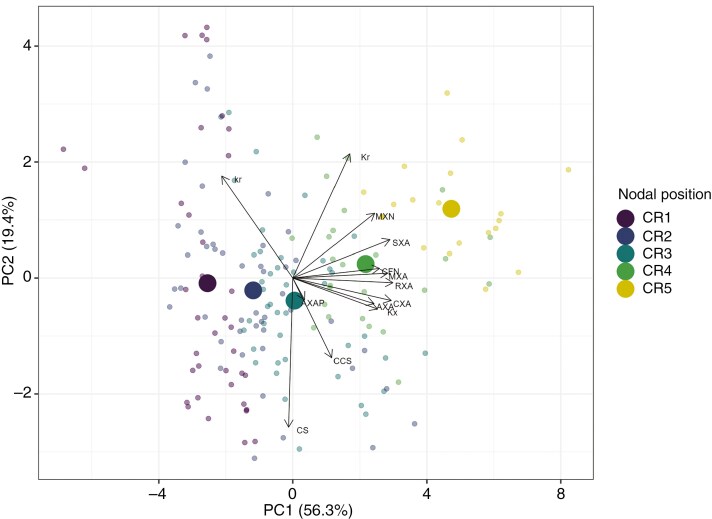
Principal component analysis of root anatomical phenes and hydraulic properties. Biplot of the first two principal components (PC1, PC2) of a principal component analysis of 10 root anatomical phenes and two hydraulic properties (determined through MECHA). Transparent points indicate scores of individual roots on these two components and opaque points represent centroids of scores per nodal position, from CR5 to CR1 (young, CR5, to old, CR1, by colour) of field-grown maize plants in drought vs. rainfed conditions. Arrows represent loadings of root anatomical phenes and hydraulic properties. The following variables were included: root cross-sectional area (RXA), stele area (SXA), cortex area (CXA), cortex:stele ratio (CS), cortical cell size (CCS), cortical file number (CFN), metaxylem number (MXN), total metaxylem area (MXA), aerenchyma area (AXA), aerenchyma per cent (AXAP), radial hydraulic conductivity (*k*_r_), radial hydraulic conductance (*K*_r_) and axial hydraulic conductance (*K*_x_).

The interaction between node of origin, genotype and drought treatment significantly impacted the development of overall root anatomy (Table 2), with nodal position showing the strongest effect (see effect size Table 2; Fig. 5).

**Table 2.** PERMANOVA results on integrated root anatomy including the following factors: nodal position (R), genotype (G), treatment (T) and their interactions. PERMANOVA was computed on Euclidean distances calculated on log- and *z*-transformed data. The following variables were included: root cross-sectional area, stele area, cortex area, cortex:stele ratio, cortical cell size, cortical file number, metaxylem number, total metaxylem area, aerenchyma area and aerenchyma per cent. Significant factors [*P*(perm) ≤ 0.05] are shown in bold.

Source	d.f.	SS	MS	Pseudo-*F*	*P*(perm)	Perms	Effect size
**R**	**4**	**867.40**	**216.85**	**13.92**	**0.0002**	**9948**	**2.49**
**G**	**3**	**127.82**	**42.61**	**12.02**	**0.0001**	**9936**	**1.01**
T	1	8.90	8.90	0.59	0.6943	7893	−0.28
**R:G**	**12**	**194.53**	**16.21**	**4.57**	**0.0001**	**9888**	**1.22**
R:T	4	48.74	12.19	1.31	0.2630	9936	0.42
**G:T**	**3**	**45.59**	**15.20**	**4.29**	**0.0002**	**9961**	**0.78**
**R:G:T**	**12**	**115.60**	**9.63**	**2.72**	**0.0001**	**9900**	**1.19**

Since the effects of genotype and drought treatment on the phene-independent integrated root anatomy (i.e. multivariately considering all phenes that define root anatomy collectively) interacted significantly with node of origin, we investigated their effect in detail by pairwise comparisons. The drought treatment effect on the phene-independent integrated root anatomy across genotypes was most consistent for roots emerging from the second node (CR2; Supplementary Data Table S2). Genotypic differences in the effect of the drought treatment on the phene-independent integrated root anatomy were apparent but followed no systematic pattern (Table S3) and are not specific to either landraces or modern cultivars, neither individually nor in interaction with other tested factors (Table S1).

**Fig. 6.**
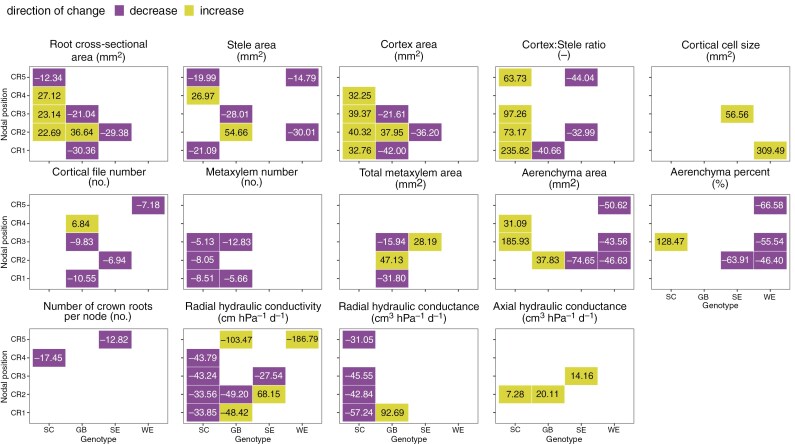
Mean relative difference (%) in anatomical and hydraulic phenes from control to drought conditions per nodal position and genotype. Colours indicate a significant difference in pairwise tests of drought treatments (*P* ≤ 0.05). Purple indicates a decrease under drought conditions compared to control conditions, while green indicates an increase under drought conditions compared to control conditions.

The change in the expression of individual phenes comparing drought and control conditions per genotype was largely inconsistent across nodal positions, i.e. not unidirectional across nodal positions per genotype or across genotypes per node (Fig. 6).

**Fig. 7.**
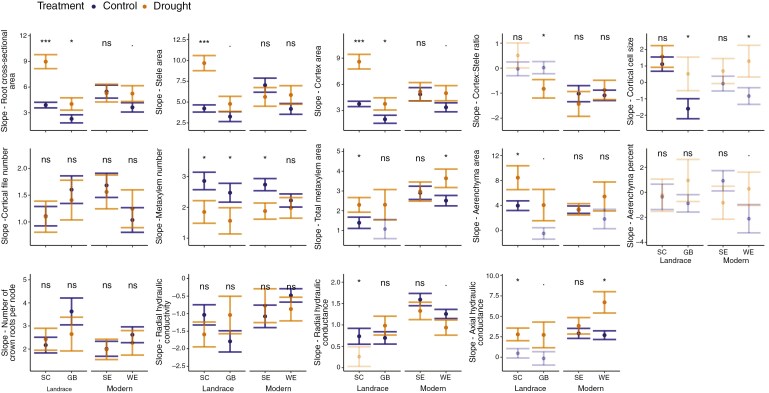
Slope of the GLM (Fig. 2) characterizing the change in a root anatomical phene across nodes (from CR1 to CR5) between drought treatments per genotype as an integrative drought response indicator. Transparent colours indicate that a phene did not change significantly across nodes (i.e. with age, Fig. 2, GLM *P* > 0.05). Significance levels of treatment differences are given for each root anatomical phene per genotype [*P* <0.1^.^, 0.05*, 0.001**, 0.0001***, not significant (>0.1), ns].

To get an integrated understanding of changes in root anatomical phenes and hydraulic properties between drought treatments per genotype over the progression of drought stress, we analysed the change of a respective trait between consecutive shoot nodes (i.e. from the oldest root CR1 to the youngest root CR5) per genotype and treatment (Fig. 2) and compared the resulting slopes between treatments per genotype (Fig. 7). The number of crown roots increased two-fold on average from the oldest to the most recently developed node (Supplementary Data Fig. S8). Root cross-sectional area increased approximately eight-fold on average from the oldest to the youngest roots (Fig. S8). Consequently, stele, and cortex area, cortical file number, metaxylem number and area, aerenchyma area, and radial and axial hydraulic conductance increased as well, while radial hydraulic conductivity decreased (Figs S8 and S10). Notably, most of those traits did not simply covary with increasing root cross-sectional area but followed a different allometric trajectory (i.e. the relative difference between drought treatments in a change in phene with increasing root cross-sectional area; Anfodillo *et al.*, 2016) under drought (Fig. S9).

Comparing drought treatments, the cross-sectional area of the root, stele, cortex, metaxylem and aerenchyma, as well as axial hydraulic conductance, increased considerably more strongly (i.e. steeper slope) with root age under drought conditions than under control conditions across most genotypes (Fig. 7). Metaxylem number increased less strongly under drought conditions compared to under control conditions. Cortex:stele ratio, cortical file number, the number of crown roots per node, and radial hydraulic conductivity and conductance mostly did not show a directed differential response to the drought treatment.

**Fig. 8.**
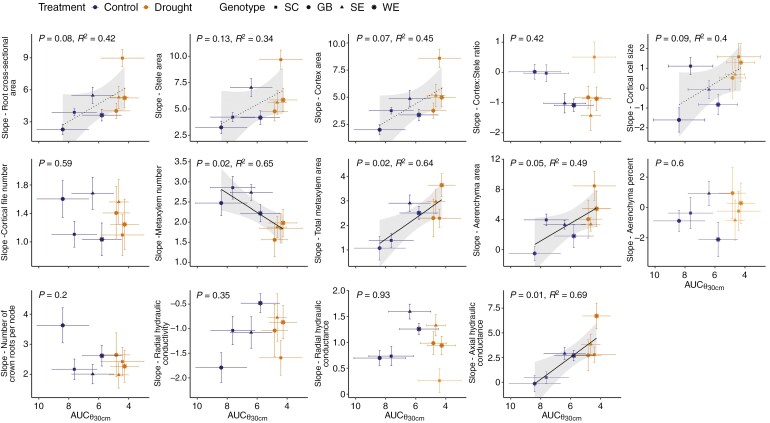
Relation between the slope characterizing the change in a root anatomical phene across nodes (from CR1 to CR5, Fig. 2) and the area under the curve of soil water content at 30 cm soil depth over time (AUC_θ30cm_, Fig. 3B). Solid lines indicate a relation between the change of a phene across nodes and soil drought with *P* ≤ 0.5, while dashed lines indicate trends in this relation until *P* ≤ 0.15. Transparent coloured symbols indicate that a phene did not change significantly across nodes.

In addition to comparing the drought treatments as two distinct categories, we also considered the drought intensity that each genotype–treatment combination experienced (Fig. 8). Under drier conditions (indicated by smaller AUC_θ30cm_ values), the areas of root, cortex, cortical cells, metaxylem and aerenchyma as well as axial hydraulic conductance increased significantly more strongly with root age compared to control conditions. By contrast, metaxylem number increased less strongly with root age under drought conditions. The same trends, albeit mostly non-significant, were observed when relating the change in root anatomical phenes with age to the soil water potential time series (Supplementary Data Fig. S10). Root anatomical phenes showed no response to water content dynamics at 60 cm soil depth (Fig. S11).

## DISCUSSION

Maize roots exhibit plastic responses to low moisture availability that probably affect their performance under drought stress (Schneider and Lynch, 2020). While maize is known to have wide genotypic variation for root anatomical phenes and their responses to drought for specific nodal positions (Schneider *et al.*, 2020), anatomical responses to drought have not been studied across multiple nodes before. This study describes how root anatomical phenes of four field-grown maize varieties respond to progressive soil drought and related changes in root hydraulic properties.

### Node order matters greatly for the evaluation of root anatomical responses

Drought responses of anatomical and hydraulics phenes differed greatly between crown roots that emerged from different nodes within and across genotypes. This is in line with Yang *et al.* (2019), who have already demonstrated the critical impact of node order on root anatomical adaptations to abiotic stress in the context of nitrogen limitations. Here, we demonstrate that critically considering the node of origin is also crucial in the context of soil water limitations.

Specifically, the response of root anatomical phenes to progressive drought stress was not consistently significant or even unidirectional across nodal positions within the same genotype or between genotypes at the same node of origin. Only crown roots originating from the second node (CR2) exhibited a consistently significant difference in overall root anatomy between drought treatments across genotypes (Supplementary Data Table S2). However, this was not necessarily uniform across genotypes at the individual phene level, even within the CR2 roots. For example, the root cross-sectional area of CR2 roots increased more strongly with age under drought for genotypes SC and GB, increased more strongly with age under controlled conditions for SE, and showed no significant difference between treatments for WE (Fig. 6).

On the one hand, this finding may suggest that roots of different nodes of origin could be governed by distinct genetic control (Hochholdinger *et al.*, 2004; Yang *et al.*, 2019; [Bibr CIT0076][Bibr CIT0078], 2023), a hypothesis that remains to be systematically investigated. On the other hand, the observed variations in node- and genotype-specific phene expression between drought treatments highlight the spatio-temporal specificity of root development in response to its surrounding microenvironment (Clément *et al.*, 2022), exemplifying root type- and genotype-specific phenotypic plasticity (Sultan, 2000). In other words, roots originating from different nodes, or even roots originating from the same node across different genotypes, may have experienced varying levels of drought intensity and dynamics during their development, leading to different degrees of deviation from the control conditions.

Those variations in drought intensity/dynamics experienced by roots originating from different nodes can partly be attributed to (1) spatial and temporal variations in soil water availability (Jiang and Whalen, 2025). For instance, water availability was comparable between drought treatments at a soil depth of 60 cm (Fig. 3B) and at the beginning of the season in June (Supplementary Data Fig. S6). Roots that developed under wetter conditions – whether in soil regions with higher water availability or during periods of greater water availability (e.g. roots that emerged before the shelters were installed) – may not exhibit adaptations to drought. Additionally, node- and genotype-specific differences in the response to drought might be related to (2) genotype- and potentially node-specific growth-rate differences. While it is generally assumed that maize plants start developing crown roots around 2 weeks after sowing and cease initiating new crown roots upon flowering (Hochholdinger, 2009), experimental evidence addressing genotypic and node-specific variability in these growth dynamics is limited. Together, our results suggest that knowledge of soil water spatio-temporal dynamics and of genotype- and node-specific root developmental patterns would be crucial to target roots that experienced comparable drought conditions if only one node is targeted for anatomical comparisons. In practice, root anatomical phenotypes are preferably evaluated on either the second or the fourth shoot node (Chimungu *et al.*, 2014*a*, *b*, 2015*b*; Klein *et al.*, 2020; Schneider *et al.*, 2020). In some studies, a specific node is not even targeted (Zhu *et al.*, 2010; Hazman and Kabil, 2022). Considering our findings, evaluating only one node may not be sufficient to characterize the response of root anatomical phenes to drought, especially in experiments under field conditions where uniform soil water distribution – both temporally and spatially – cannot be assumed. If evaluating changes in phene expressions between soil moisture treatments on roots of one node only, soil water dynamics and root growth should be continuously monitored to increase the chances of targeting comparable roots that experienced the desired treatment. Based on our data, roots of second node crown roots might be a better target than other roots, although this is expected to vary in a different experimental setup.

Intriguingly, clear treatment-specific patterns of root anatomical adaptations became evident when analysing the changes in individual root anatomical phenes relative to the node of origin, i.e. relative root age, as integrated over the whole root system.

### Multiple integrated phenes shape root hydraulics under limited soil water availability

The majority of the investigated root anatomical phenes showed a (hyper)allometric relationship with increasing root cross-sectional area from older to younger roots (i.e. due to increased shoot growth and demand for water and nutrients, Yu *et al.*, 2015). However, these relationships followed distinct trajectories (Anfodillo *et al.*, 2016) under control vs. drought conditions (Supplementary Data Fig. S9). Under drier conditions, the cross-sectional area of the root, cortex, cortical cells, metaxylem and aerenchyma increased more strongly with relative root age, i.e. between nodes, compared to control conditions (Figs 7 and 8). By contrast, the number of metaxylem vessels tended to increase moderately under drought compared to control conditions (Figs 7 and 8).

Qualitatively concluding from changes in one phene to effects on root hydraulics is challenging due to synergies and trade-offs between simultaneously changing traits. For example, the less pronounced increase in the number of metaxylem vessels under drought conditions, as observed here (Figs 7 and 8), would be expected to lead to a reduced root axial hydraulic conductance. Richards and Passioura (1989) observed that wheat plants with a reduced axial hydraulic conductance (i.e. plants with narrower xylem vessels in their seminal roots) yielded 3–11 % more in dry environments. This yield improvement was attributed to enhanced sub-soil water conservation during the vegetative phase, allowing for greater water availability during anthesis (i.e. ‘water banking’). Klein *et al.* (2020) similarly found that improved ‘drought tolerance’ in maize (by them defined as a minimal reduction in vegetative biomass accumulation under water stress) was linked to a reduced axial hydraulic conductance associated with narrower xylem vessels. However, in our case, the total metaxylem area simultaneously increased significantly more steeply between consecutive nodes under drought conditions (Figs 7 and 8), resulting in a greater metaxylem area per vessel. The increase in vessel size effectively compensated for the reduced number of vessels, resulting in an overall increase in simulated axial hydraulic conductance (Fig. 8; Supplementary Data Fig. S12). This increase in axial hydraulic conductance under drier conditions observed in our study is not in line with Richards and Passioura (1989) or Klein *et al.* (2020) but with a recent study by Affortit *et al.* (2024*b*). The latter authors recently reported that a higher metaxylem area was associated with greater grain weight in pearl millet. They attributed this positive correlation to the increased (stomatal) sensitivity to soil drying that would be expected for more conductive plants (Koehler *et al.*, 2023*a*, *b*), which could contribute to water saving, eventually. Alternatively, our results may suggest that the genotypes investigated here are optimized for aggressive water acquisition in wetter environments rather than for soil water conservation in drought-prone regions (Tyree *et al.*, 1994). However, scaling this up to encompass the axial water transport capacity of the entire root system would need to consider the limiting effect that increased total root length imposes on the axial hydraulic conductance of the whole root system (Meunier *et al.*, 2017; Bouda *et al.*, 2018).

While axial hydraulic conductance provides insight into the root’s longitudinal water transport capacity, the primary hydraulic constraints on root water uptake are believed to occur along the root’s radial pathway in drying soils (Frensch and Steudle, 1989; Zwieniecki *et al.*, 2002). In our study, the significant increase in cortical width and aerenchyma area would be expected to reduce the radial conductivity (Fan *et al.*, 2007; Chimungu *et al.*, 2014*a*; Lynch *et al.*, 2014), whereas the increased xylem area would (marginally) enhance it (Heymans *et al.*, 2020). The combination of these adaptations to soil drought levelled out in their effect on the simulated radial hydraulic conductivity, resulting in the change with age being comparable between drought and control conditions (Fig. 8). Consequently, also root hydraulic conductance did not differ significantly under drought, despite the more pronounced increase in root cross-sectional area under drought. However, our quantification of radial water uptake capacity could not account for non-anatomical drought responses, such as the activity and distribution of water channelling aquaporins (Knipfer *et al.*, 2011), the formation of hydrophobic barriers through suberization and lignification (Henry *et al.*, 2012; Hazman and Brown, 2018) or the conductivity of plasmodesmata (Couvreur *et al.*, 2018), as well as for dynamic properties of the root–soil interface (Ahmed *et al.*, 2018*a*; Affortit *et al.*, 2024*a*). While the absence of drought treatment differences in rhizosheath (Supplementary Data Fig. S7C) suggests negligible rhizosphere drought adaptations, we cannot rule out drought-related changes in aquaporin activity (Shivaraj *et al.*, 2021), which are suggested to alleviate drought effects (Ahmed *et al.*, 2021). Thus, radial water uptake capacity under drought conditions may be underestimated.

Additionally, concluding from the root organ/phene scale (i.e. from axial and radial root cross-sectional hydraulic conductance) to the whole root–soil system hydraulic conductance (*K*_rs_) requires a few considerations. First, the drop in soil–root hydraulic conductance during soil drying will not only depend on limitations in root hydraulic conductance but also on a soil-texture-specific drop in soil hydraulic conductivity at the soil–root interface (van Lier *et al.*, 2006; Schröder *et al.*, 2009; Koehler *et al.*, 2022). For example, soil water potentials drop rapidly at relatively less negative soil water potentials in sandy soils compared to a more gradual drop at more negative soil water potentials in fine-textured soils such as loam. However, variations in top-soil texture between plots in our field setting are negligibly small (see same experimental setting in Tyborski *et al.*, 2024). Second, root architectural parameters were shown to shape *K*_rs_ (Bauer *et al.*, 2024). For example, Yu *et al.* (2024) have recently shown that the increase in seminal root number during maize domestication was an important driver for the increase in *K*_rs_ in seedlings. As Ahmed *et al.* (2018*b*) have demonstrated that water is mainly taken up by the crown roots in mature maize plants, we focused on the crown roots here. In this study, the increase in crown root number with age did not differ between drought treatments, indicating no treatment difference in *K*_rs_ due to the number of crown roots per node (Figs 7 and 8). Additionally, Baca Cabrera *et al.* (2024) have recently highlighted that root length relative to the proportion of conductive root segments (as a function of root age in terms of xylem vessel maturation and the development of hydrophobic barrier) shapes the development of *K*_rs_. In our case, the substantial increase in root biomass and root:shoot ratio under drought conditions (Supplementary Data Fig. S7A–B) suggests an increase in whole root system length/surface area and, hence, in whole-root system hydraulic conductance under drought, even if node-specific radial hydraulic conductivity was comparable between drought treatments (Baca Cabrera *et al.*, 2024). Combined with the relatively steeper increase in axial hydraulic conductance with age under drought, our results suggest that *K*_rs_ may have been higher under drought.

An increase in whole-root system hydraulic conductance under drought is not typically observed (although reported in Zhang *et al.*, 1995) but could support the earlier proposition that the tested genotypes lack a conservative adaptation strategy to drought-stressed environments. Considering that the varieties selected for this study (1) originate from and are adapted to the relatively well-watered environments of Germany (Purushothaman *et al.*, 2013) and (2) were grown in highly conductive soil here, an aggressive water-use strategy may indeed have been more advantageous for optimizing yield (Sadok *et al.*, 2019). To verify this, water use responses to drought would need to be studied, as in Affortit *et al.* (2024*b*). This highlights the complex trait × environment interactions.

However, as soil drought becomes an increasingly frequent phenomenon in the course of climate change, the importance of soil water conservation is expected to increase. Accordingly, strategies to reduce daily water use, e.g. achieved by lowering root hydraulic conductance, have shown great potential for enhancing the probability of yield gains (Sinclair, 2018). Phenes that contribute to a desired strategy might, therefore, be interesting for target-environment-specific root ideotype breeding, which is discussed as being a promising method for optimizing water use in a particular environment (Lynch, 2013, 2019). According to Klein *et al.* (2020), an ideotype may consist of any number of phenotypic characteristics to optimize performance in a given environment, resulting in a vast and complex landscape of target-environment-specific integrated phenotypes. As demonstrated in our study, successfully identifying such ideotypes additionally requires considering root anatomical phenes at several nodal positions.

## CONCLUSION

We highlight the complex and node-specific anatomical responses of maize roots to progressive soil drought, underscoring the importance of considering node of origin in evaluating drought responses of anatomical phenes. The significant variability in root anatomical phene responses to soil drought among different genotypes and nodal positions indicates that a one-size-fits-all approach to root sampling may overlook critical insights into drought adaptation. Notably, the second node crown roots showed the most consistent and significant anatomical changes in response to drought, suggesting that they may be a valuable focus for future studies and breeding programmes aimed at enhancing drought tolerance.

Further, we found that multiple integrated phenotypes, overlapping each other in their synergistic or antagonistic effects on the root’s water uptake and transport capacity, shaped root cross-section hydraulic properties in a qualitatively unpredictable way. Concluding from one phene to one function is therefore not functional against the array of background phenotypes with which it may potentially interact. Hence, we strongly support Klein *et al*. (2020) in their statement: ‘Addressing integrated phenotypes, as opposed to a single phene, will be an invaluable tool for breeders to develop crop varieties suited to a given agroecosystem’, and want to additionally emphasize the usefulness of structural–functional models in translating the combination of phenes into global parameters (i.e. whole root system hydraulic conductance) that matter for water use (e.g. GRANAR: Heymans *et al.*, 2020; MECHA: Couvreur *et al.*, 2018; CPlantBox: Giraud *et al.*, 2023).

## SUPPLEMENTARY DATA

Supplementary data are available at *Annals of Botany* online and consist of the following.

Fig. S1: Map of the complete field layout. Fig. S2: Daily time-series of temperature and relative humidity under drought and control conditions. Fig. S3: Exemplary maize root cross-section (SE control CR3). Fig. S4: Time series of soil water content at 30 cm soil depth. Fig. S5: Time series of soil water content at 60 cm soil depth. Fig. S6: Time series of soil water potential at 30 cm soil depth. Fig S7: Belowground plant performance between drought treatments in terms of root dry biomass, root:shoot ratio and specific rhizosheath mass. Fig. S8: Expression of root anatomical traits dependent on the node of origin, i.e. age per drought treatment and per genotype. Fig. S9: Allometric relations between individual root phenes with the increase in root cross-sectional area between roots from consecutive shoot nodes. Fig. S10: Relation between the slope characterizing the change in a root anatomical trait with age and the area under the curve of soil water potential at 30 cm soil depth over time. Fig. S11: Relation between the slope characterizing the change in a root anatomical trait with age and the area under the curve of soil water content at 60 cm soil depth over time. Fig. S12: Dependence of axial hydraulic conductance on total metaxylem area and the number of metaxylem vessels. Table S1: PERMANOVA results on root anatomical traits including the following factors: root node, landrace vs. modern variety, drought treatment, and genotype nested in landrace vs. modern variety. Table S2: Results of pairwise PERMANOVA on Euclidean distances comparing levels of the factor drought treatment within levels of the factors genotype and root node. Table S3: Results of pairwise PERMANOVA on Euclidean distances comparing levels of the factor genotype within levels of the factors drought treatment and root node.

mcaf075_suppl_Supplementary_Figures_S1-S12_Tables_S1-S3
