# Supplementary material for: Node of origin matters: comparative analysis of soil water limitation effects on nodal root anatomy in maize (Zea mays)
Source: Ann Bot. 2025 May 15;136(5-6):1031–46. doi: 10.1093/aob/mcaf075 (PMC12682840; doi:10.1093/aob/mcaf075)
Supplement: mcaf075_suppl_Supplementary_Figures_S1-S12_Tables_S1-S3 [file mcaf075_suppl_supplementary_figures_s1-s12_tables_s1-s3.docx]

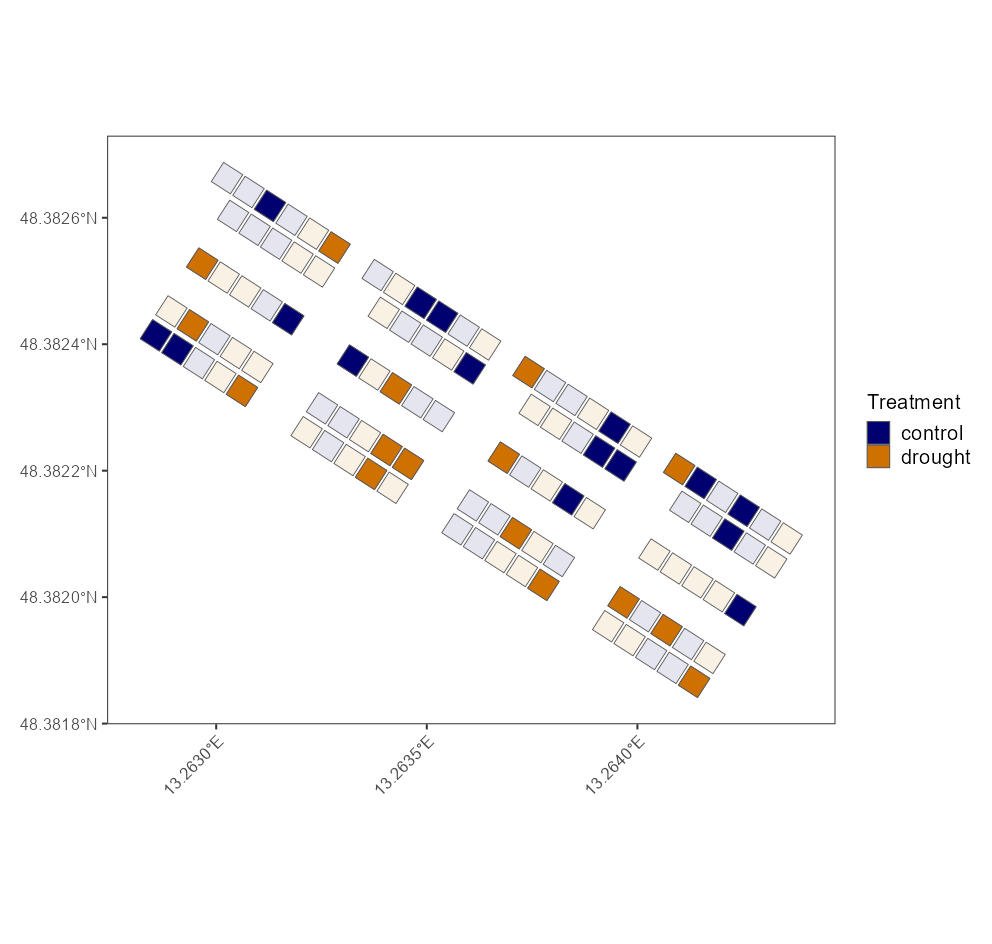


Fig. S1: Map of the complete field layout. Each square represents a distinct plot. Opaque squares denote plots selected for root anatomical analyses, while transparent squares indicate plots where root anatomical analyses were not conducted. Plots shaded in orange were covered by rain-out shelters (= drought), while blue-shaded plots were rainfed (= control).


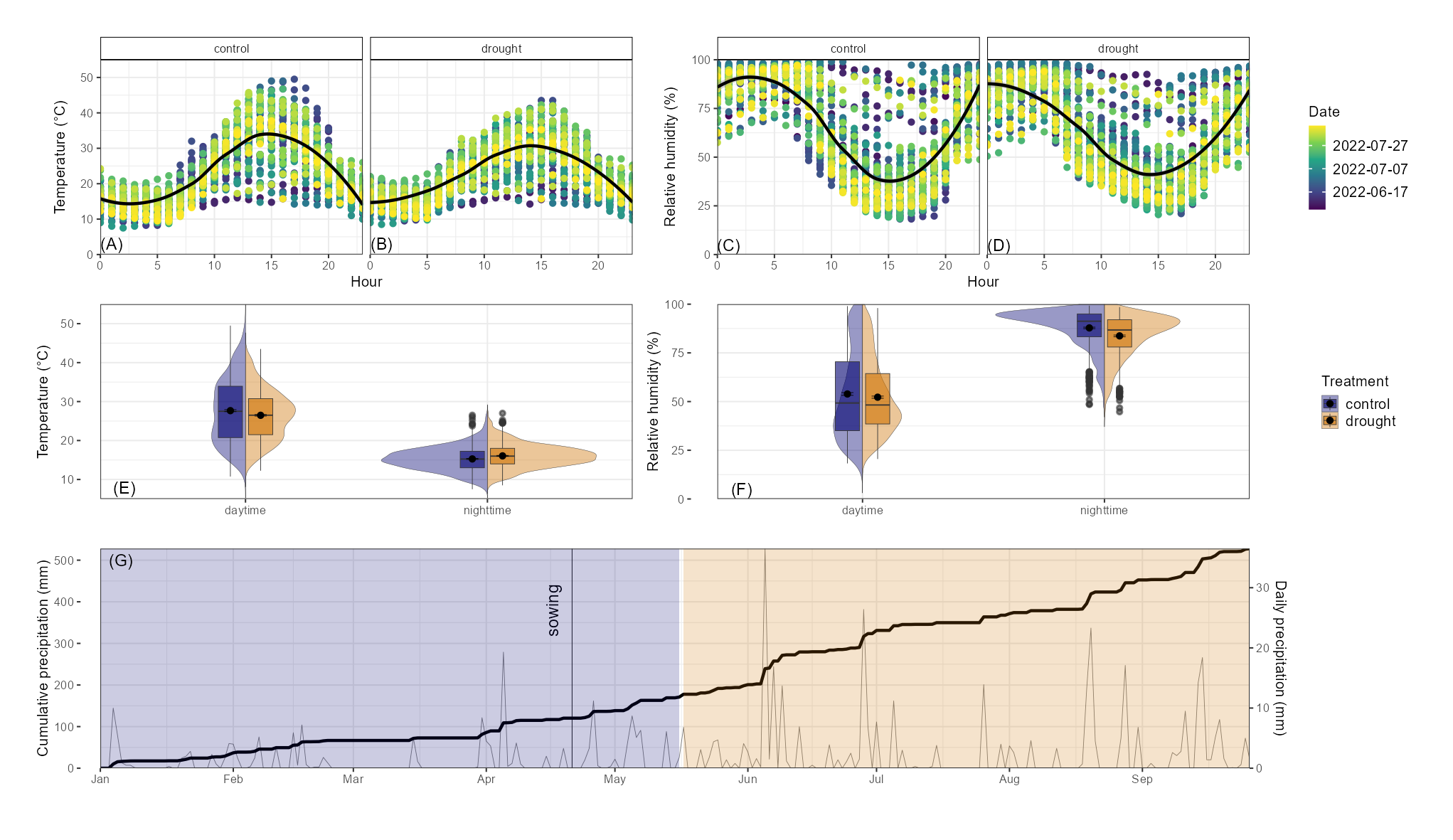


Fig. S2: Daily time-series of temperature (A,B) and relative humidity (C,D) under drought (B,D) and control (A,C) conditions. Daytime (07:00-21:00) and nighttime (22:00-06:00) averages over the whole period between drought-treatments for temperature (E) and relative humidity (F). Cumulative precipitation from the beginning of the year 2022 (left y-axis) and daily precipitation (right y-axis) until the completion of the harvest (G). Blue-shaded background indicates time before the rainout shelter set up. Orange-shaded background indicates time after rainout shelter set up. Data was accessed through Meteostat (meteostat.net) from the nearest weather station in Pocking (lat. 48.395245, long. 13.313896, 4.0 km east-north-east of the field location).


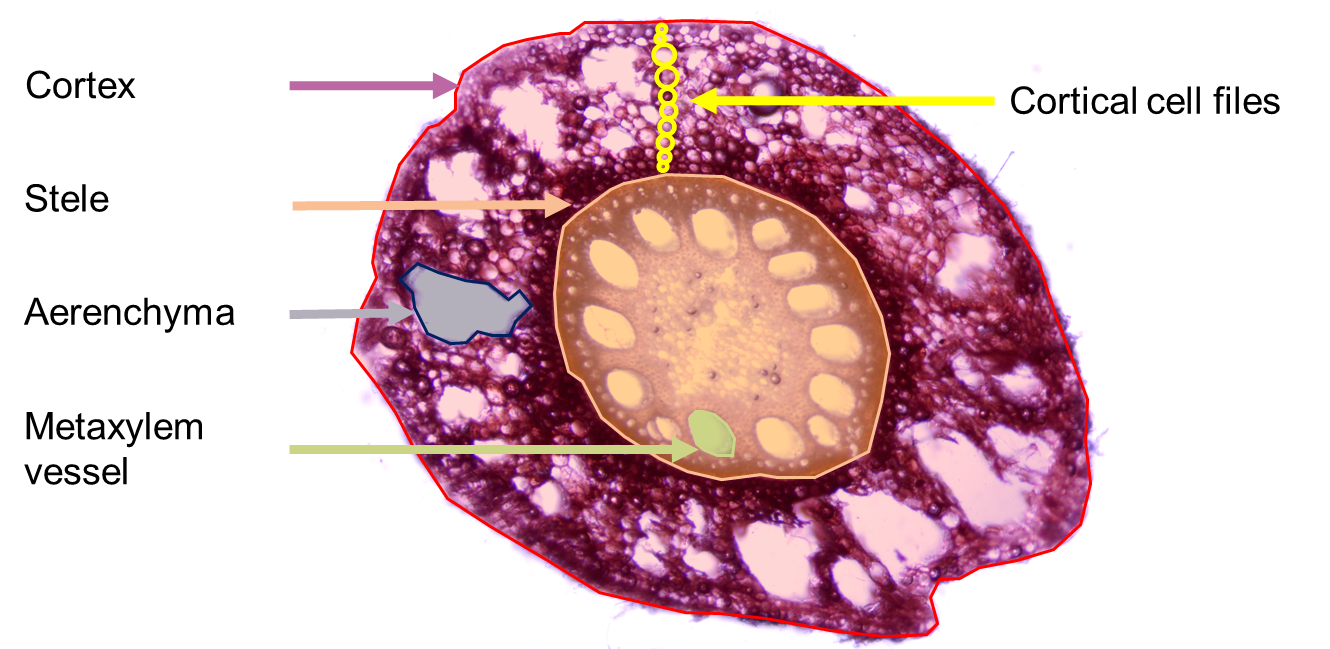


Fig. S3: Exemplary maize root cross-section (SE control CR3). The outermost boundary of the root represents the root cross-sectional area. The purple-shaded area indicates the cortex, while the orange-shaded area represents the stele. The blue-shaded area corresponds to aerenchyma, and the green-shaded area depicts the metaxylem area of a single vessel. The yellow circles highlight the cortical cell files. Quantification using ImageJ was performed according to Yang et al. (2019 - Supplementary Fig. S2).


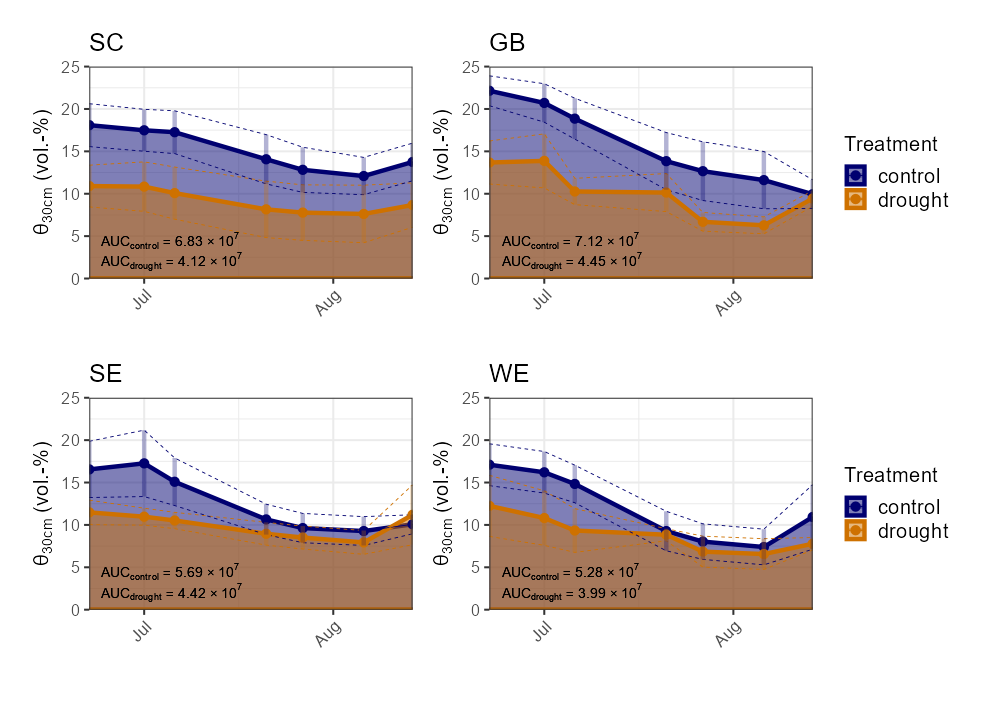


Fig. S4: Time series of soil water content in 30 cm soil depth (θ_30cm_). The dots represent mean θ_30cm_ across replicate plots per genotype, drought-treatment, and day. The dashed lines represent the standard error (SE) of mean θ_30cm_ per day. The shaded area represents the area under the curve (AUC) per treatment (control, drought) ± SE. AUCs summarizes the soil water content time series in terms of magnitude and temporal dynamics. SC, GB, SE, and WE are the four different genotypes used in this study.


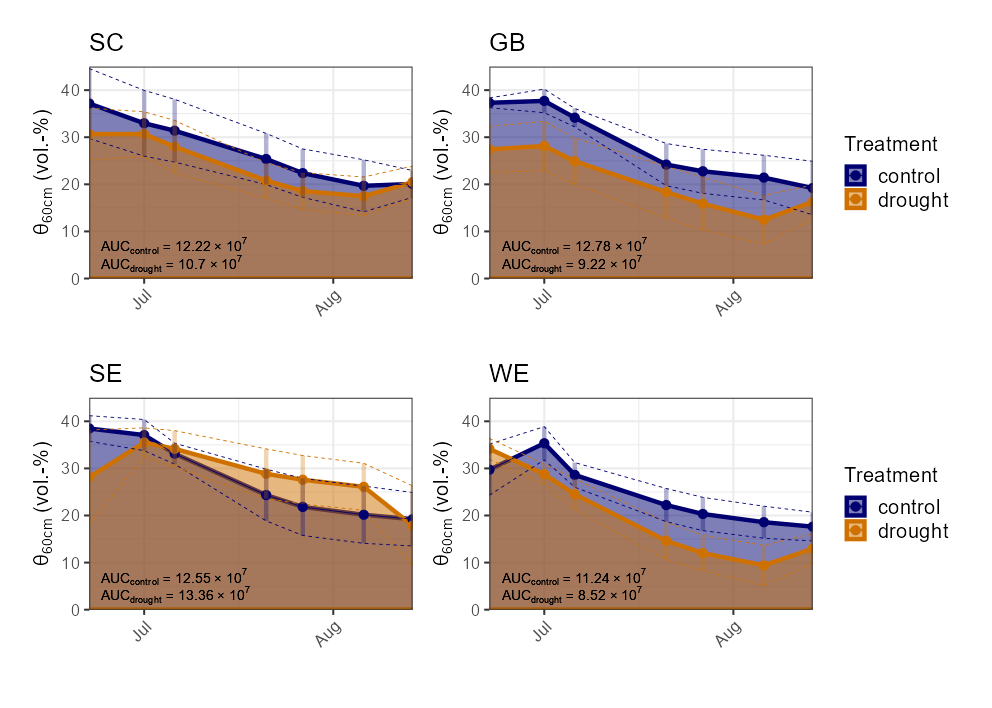


Fig. S5: Time series of soil water content in 60 cm soil depth (θ_60cm_). The dots represent mean θ_60cm_ across replicate plots per genotype, drought-treatment, and day. The dashed lines represent the standard error (SE) of mean θ_60cm_ per day. The shaded area represents the area under the curve (AUC) per treatment (control, drought) ± SE. AUCs summarizes the soil water content time series in terms of magnitude and temporal dynamics. SC, GB, SE, and WE are the four different genotypes used in this study.


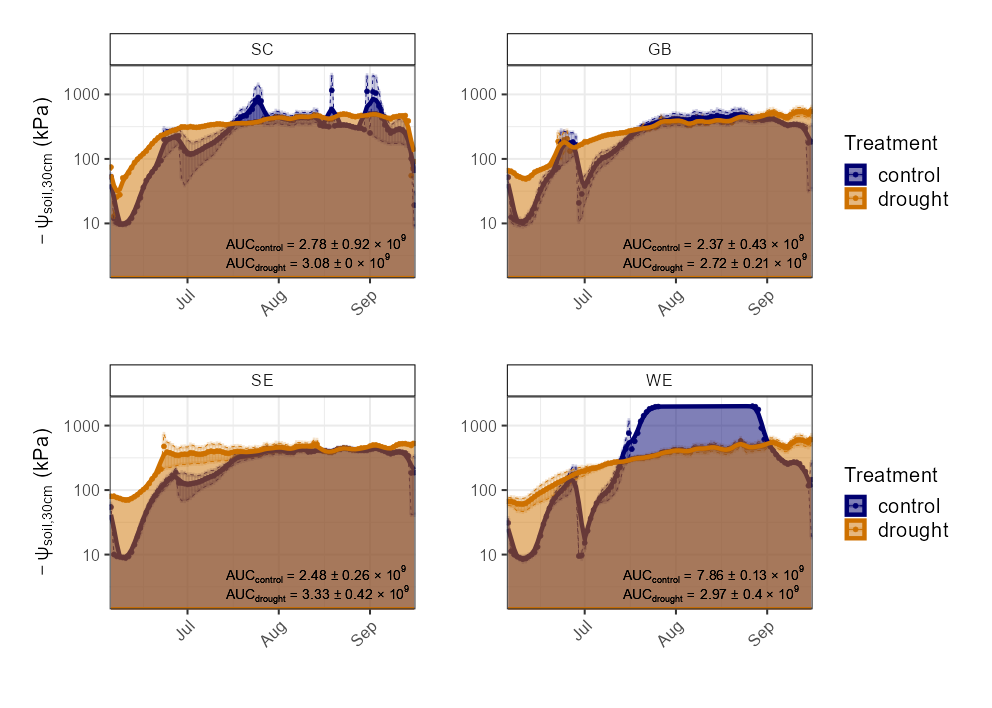


Fig. S6: Time series of soil water potential in 30 cm soil depth (ψ_soil,30cm_). The dots represent mean ψ_soil,30cm_ across replicate plots per genotype, drought-treatment, and day. The dashed lines represent the standard error (SE) of mean ψ_soil,30cm_ per day. The shaded area represents the area under the curve (AUC) per treatment (control, drought) ± SE. AUCs summarizes the soil water potential time series in terms of magnitude and temporal dynamics. SC, GB, SE, and WE are the four different genotypes used in this study.


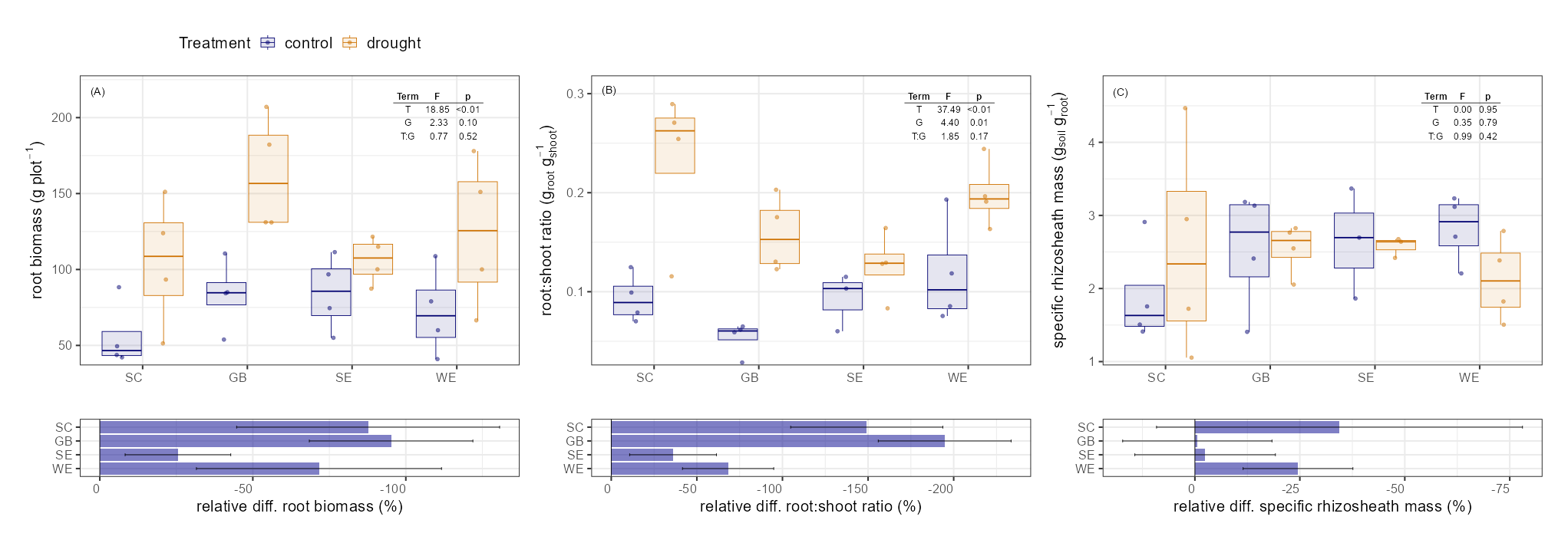


Fig S7: Belowground plant performance between drought-treatments in terms of root dry biomass (A), root:shoot ratio (B), and specific rhizosheath mass (C). Effects of drought-treatment (T), genotype (G) and their interaction (T:G) on belowground plant performance based on a linear model are indicated in the upper right corner.


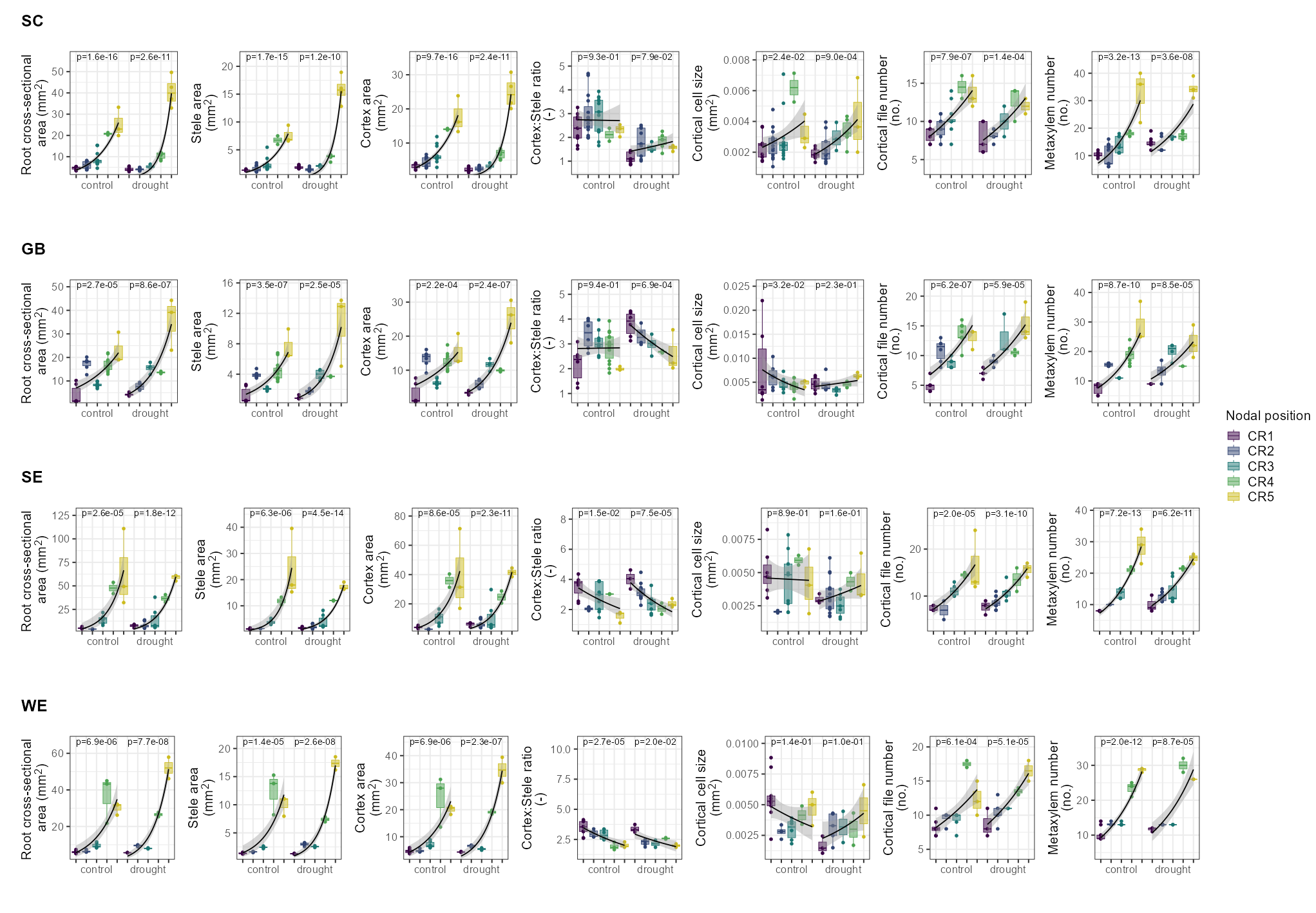


Fig. S8: Expression of root anatomical traits in dependence on the node of origin, i.e., age (from the oldest root – CR1 to the youngest root – CR5) per drought-treatment and per genotype (SC, GB, SE, WE). The black line represents the fit of a generalized linear model (GLM, with a log link function, allowing for exponential relationships). The p-value << 0.05 indicates that the glm describes the data significantly.


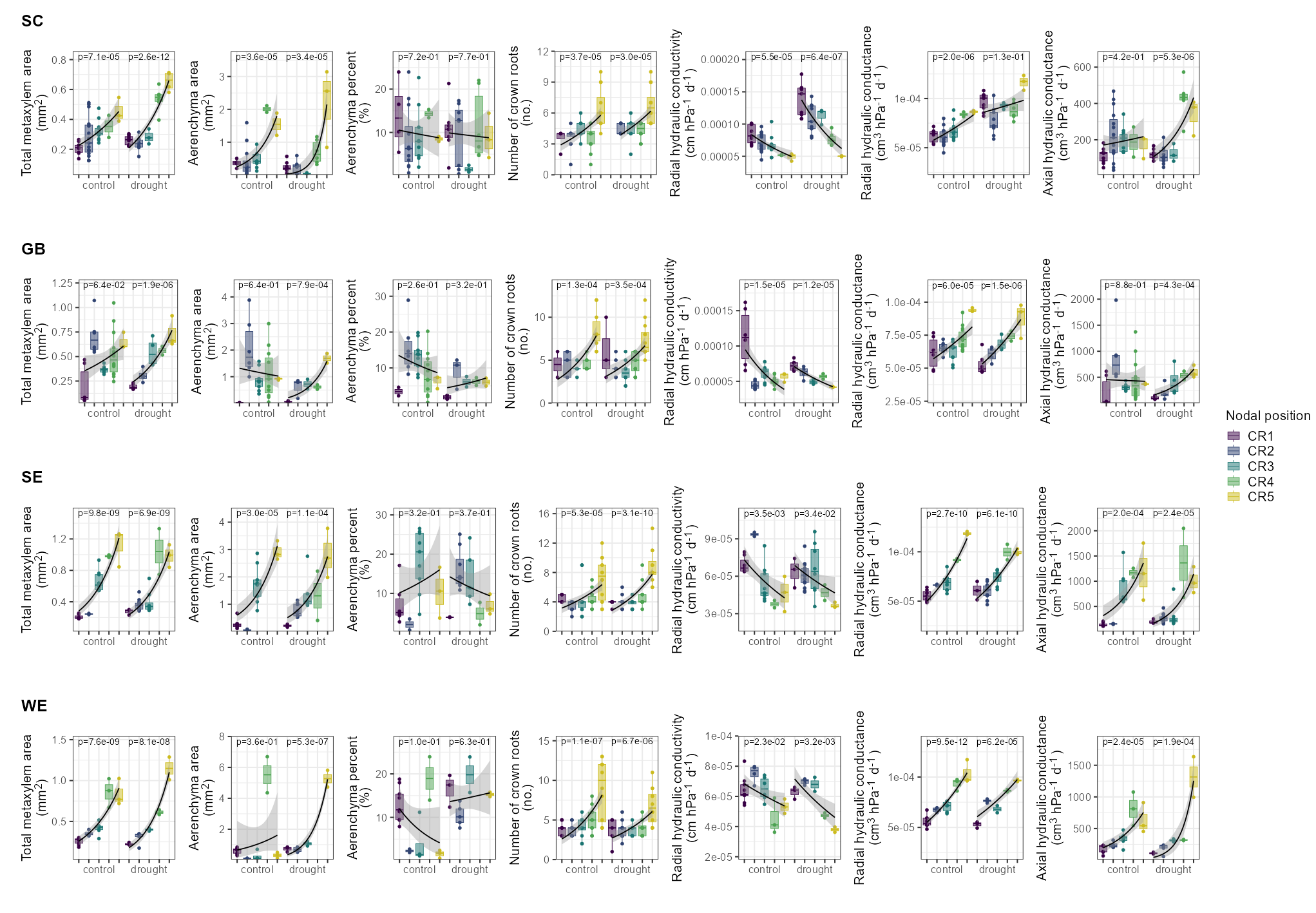


Fig. S8 - continuation: Expression of root anatomical traits in dependence on the node of origin, i.e., age (from the oldest root – CR1 to the youngest root – CR5) per drought-treatment and per genotype (SC, GB, SE, WE). The black line represents the fit of a generalized linear model (GLM, with a log link function, allowing for exponential relationships). The p-value << 0.05 indicates that the glm describes the data significantly.


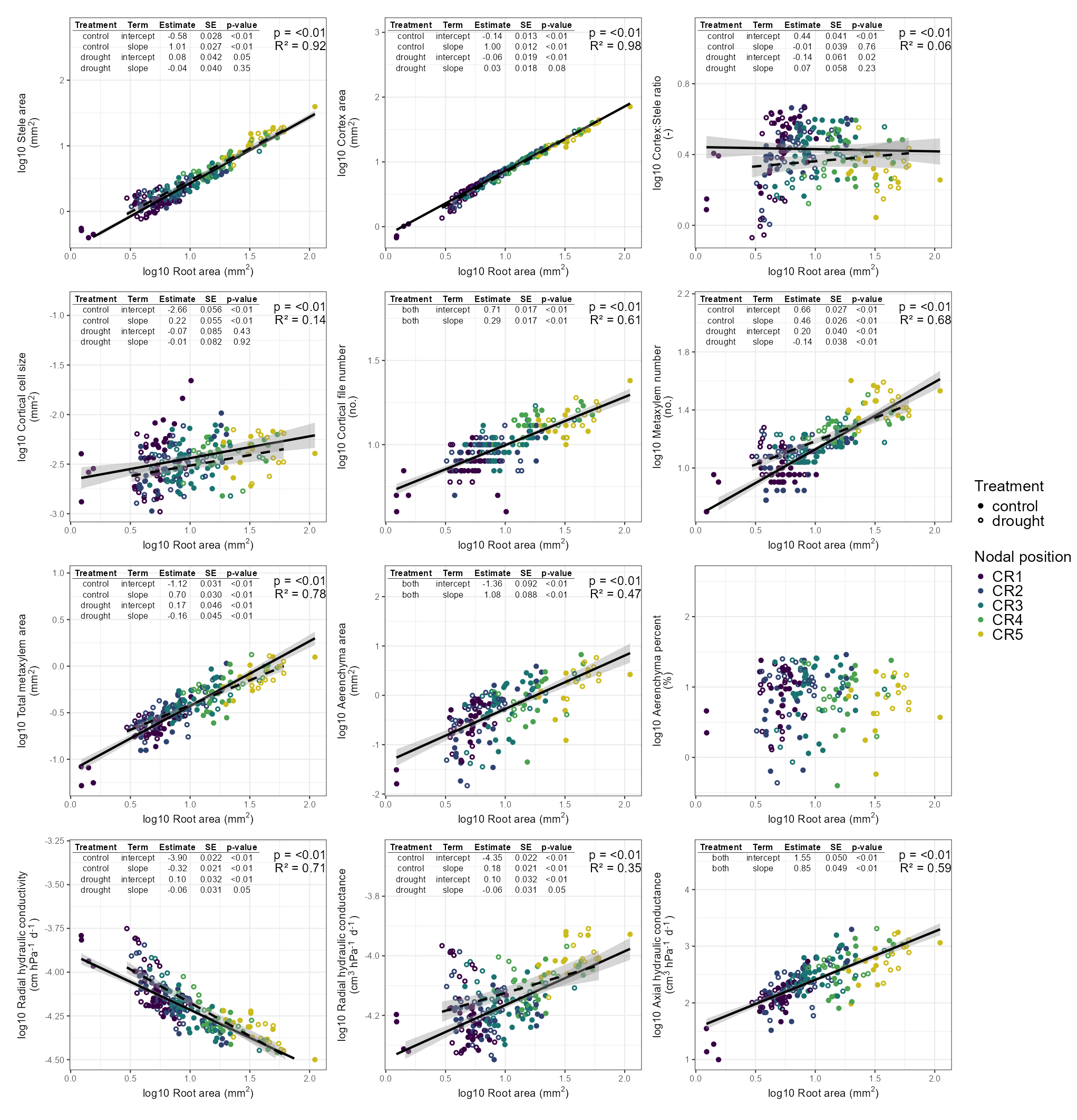


Fig. S9: Allometric relations between individual root phenes with the increase in cross-sectional root area between roots from consecutive shoot nodes (CR1-CR5, i.e., root age as indicated by colors). The linear regressions represent the allometric trajectories per drought treatment. The absence of a regression line indicates that there was no directed change in the respective phene with increasing cross-sectional root area. One solid regression line indicates that the allometric trajectory did not differ between drought treatments. Two regression lines indicate that the allometric trajectories differed between drought treatments (control= solid line, drought= dashed line). Data were log10-transformed to achieve linear relations. Via building linear models per phene with and without considering interactions with the drought treatment, we assessed whether the allometric trajectories differed between drought treatments. The models were compared by an ANVOA. If the model considering the interaction performed better (ANOVA p-value ≤ 0.05), we considered the allometric trajectories to have differed between drought treatments and listed the coefficient estimates per treatment. Positive estimates for intercept and slope for the drought treatment indicate higher values compared to the control treatment, while negative estimates indicate lower values. P-values ≤ 0.05 indicate that slope or intercept for the drought treatment differed significantly compared to the control. If the model not considering the interaction performed better, we considered that the allometric trajectories did not differed between drought treatments and applied one linear regression over both treatments. R^2^ and p in the upper right corner indicate the overall model performance.


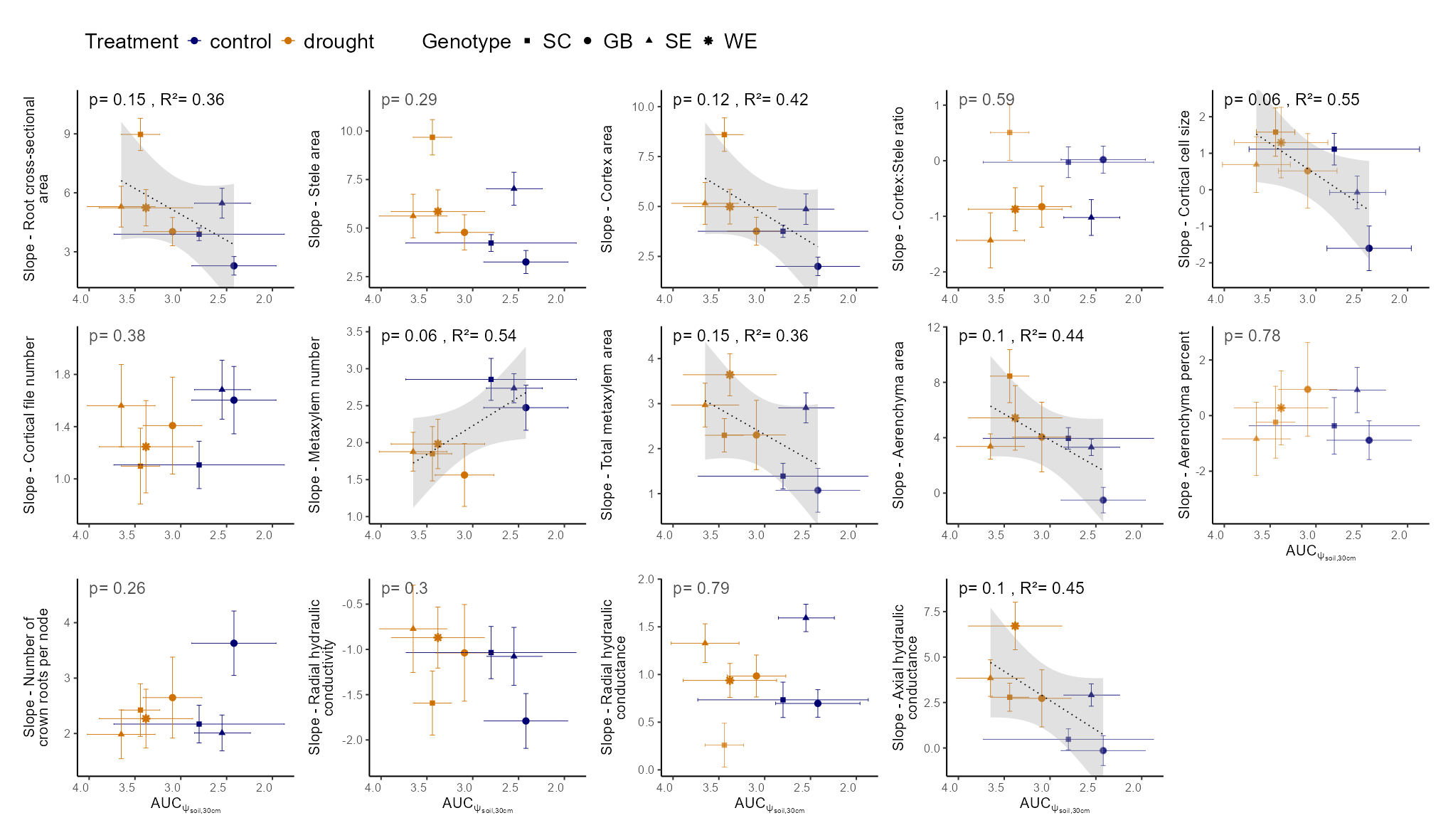


Fig. S10: Relation between the slope characterizing the change in a root anatomical trait with age (from CR1-CR5, Fig. 8) and the area under curve of soil water potential in 30 cm soil depth over time (AUC Ψ_soil,30cm_, Fig. 5C). Solid lines indicate a relation between the change of a phene with age and soil-drought with p-values ≤ 0.5, while dotted lines indicate trends in this relation until p ≤ 0.15. Transparent colored symbols indicate that a phene did not significantly change with age.


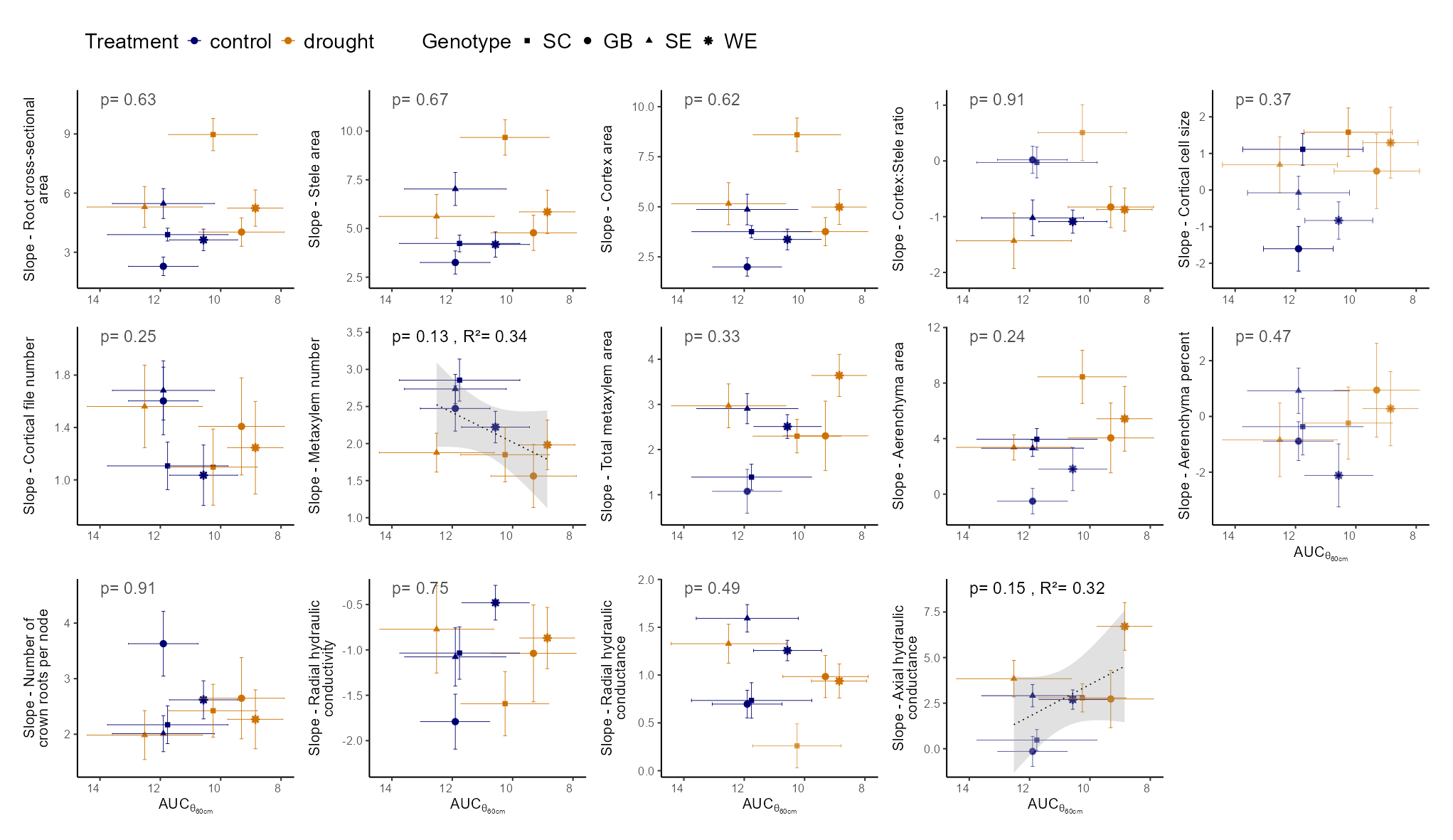


Fig. S11: Relation between the slope characterizing the change in a root anatomical trait with age (from CR1-CR5, Fig. 8) and the area under curve of soil water content in 60 cm soil depth over time (AUC_θ60cm_, Fig. 5C). Solid lines indicate a relation between the change of a phene with age and soil-drought with p-values ≤ 0.5, while dotted lines indicate trends in this relation until p ≤ 0.15. Transparent colored symbols indicate that a phene did not significantly change with age.


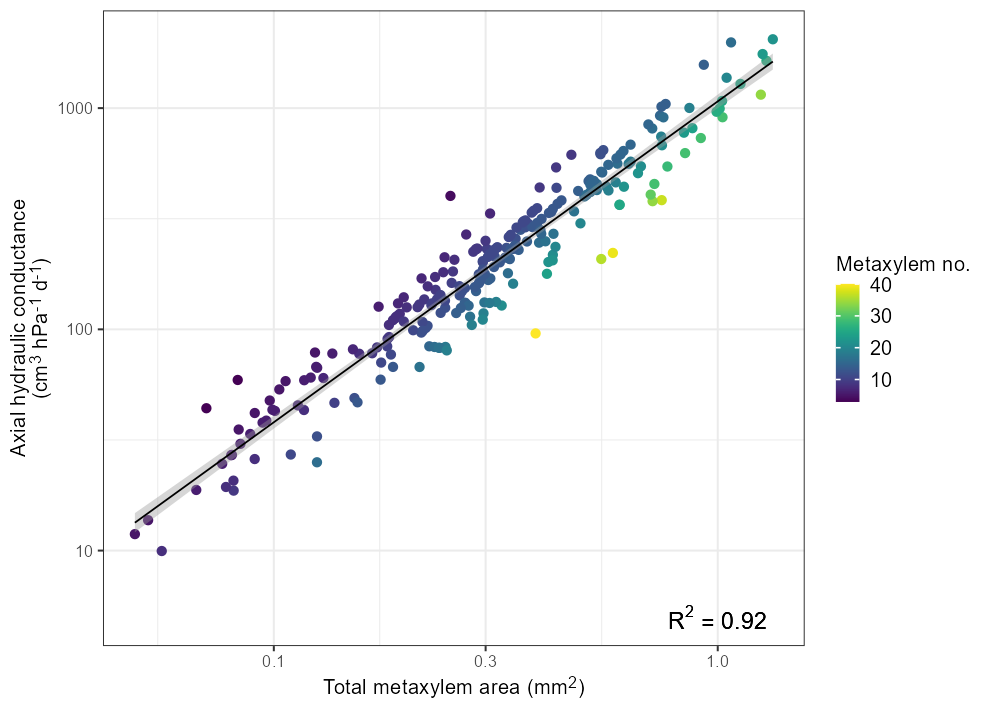


Fig. S12: Dependence of the axial hydraulic conductance (Kx) on total metaxylem area (MXA) and the number of metaxylem vessels (MXN).

Table S1: PERMANOVA results on root anatomical traits including the following factors: root node (R, fixed), landrace vs. modern variety (‘breeding era’, BE, fixed), drought-treatment (T, fixed), and genotype nested in landrace vs. modern variety (G(BE), random). PERMANOVA was computed on Euclidean distances calculated on log- and z-transformed data. Due to the low number of possible unique permutations for the factor landrace vs. modern variety (BE), we consider the P-values generated by Monte-Carlo testing (P(MC)) more meaningful here. The following variables were included: root surface area, stele surface area, cortex surface area, cortex:stele-ratio, cortical cell size, cortical file number, metaxylem number, total metaxylem surface area, aerenchyma surface area, and aerenchyma percent. Significant factors (P(perm) ≤ 0.05) are shown in bold.

| **Source** | **df** | **SS** | **MS** | **Pseudo-F** | **P(perm)** | **perms** | **P(MC)** | **effect size** |
| --- | --- | --- | --- | --- | --- | --- | --- | --- |
| **R** | **4** | **867.40** | **216.85** | **15.93** | **0.0005** | **9954** | **0.0001** | **2.50** |
| BE | 1 | 58.58 | 58.58 | 1.82 | 0.3339 | 6 | 0.2424 | 0.59 |
| T | 1 | 8.90 | 8.90 | 0.62 | 0.6069 | 4315 | 0.6285 | -0.27 |
| **G(BE)** | **2** | **65.93** | **32.96** | **9.30** | **0.0001** | **9940** | **0.0001** | **0.87** |
| RxBE | 4 | 87.63 | 21.91 | 1.61 | 0.1571 | 9935 | 0.1702 | 0.72 |
| RxT | 4 | 48.74 | 12.19 | 1.21 | 0.3391 | 9933 | 0.3505 | 0.36 |
| BExT | 1 | 15.91 | 15.91 | 1.10 | 0.4346 | 4337 | 0.4220 | 0.20 |
| **RxG(BE)** | **8** | **112.04** | **14.01** | **3.95** | **0.0001** | **9907** | **0.0001** | **1.11** |
| **G(BE)xT** | **2** | **29.47** | **14.74** | **4.16** | **0.0009** | **9953** | **0.0006** | **0.76** |
| RxBExT | 4 | 37.07 | 9.27 | 0.92 | 0.5334 | 9940 | 0.5465 | -0.31 |
| **RxG(BE)xT** | **8** | **82.37** | **10.30** | **2.90** | **0.0005** | **9921** | **0.0001** | **1.27** |

Table S2: Results of pairwise PERMANOVA on Euclidean distances comparing levels of the factor drought-treatment within levels of the factors genotype and root node. Significant differences between levels are shown in bold (P(perm) ≤ 0.05). In case of low numbers of possible unique permutations, we consider the P-values generated by Monte-Carlo testing (P(MC) ≤ 0.05) more meaningful here. The following variables were included: root surface area, stele surface area, cortex surface area, cortex:stele-ratio, cortical cell size, cortical file number, metaxylem number, total metaxylem surface area, aerenchyma surface area, and aerenchyma percent.

| Node order | Genotype | Treatment | t | P(perm) | perms | P(MC) |
| --- | --- | --- | --- | --- | --- | --- |
| CR5 | WE | drought, control | 3.90 | 0.0967 | 10 | 0.0109 |
| CR5 | GB | drought, control | 1.39 | 0.1992 | 7 | 0.1885 |
| CR5 | SE | drought, control | 0.89 | 0.8051 | 10 | 0.5169 |
| CR5 | SC | drought, control | 2.12 | 0.0866 | 35 | 0.0349 |
| CR4 | WE | drought, control | 1.40 | 0.2052 | 10 | 0.1954 |
| CR4 | GB | drought, control | 0.61 | 0.7750 | 120 | 0.6786 |
| CR4 | SE | drought, control | 0.78 | 1.0000 | 3 | 0.5841 |
| **CR4** | **SC** | **drought, control** | **2.54** | **0.0391** | **28** | **0.0084** |
| **CR3** | **WE** | **drought, control** | **2.64** | **0.0341** | **28** | **0.0375** |
| **CR3** | **GB** | **drought, control** | **4.04** | **0.0016** | **715** | **0.0001** |
| CR3 | SE | drought, control | 1.54 | 0.0827 | 8099 | 0.0907 |
| **CR3** | **SC** | **drought, control** | **2.45** | **0.0021** | **560** | **0.0009** |
| **CR2** | **WE** | **drought, control** | **3.63** | **0.0071** | **126** | **0.0023** |
| **CR2** | **GB** | **drought, control** | **2.72** | **0.0049** | **210** | **0.0043** |
| **CR2** | **SE** | **drought, control** | **3.11** | **0.0091** | **120** | **0.0002** |
| **CR2** | **SC** | **drought, control** | **2.06** | **0.0020** | **9914** | **0.0106** |
| **CR1** | **WE** | **drought, control** | **2.27** | **0.0232** | **220** | **0.0175** |
| CR1 | GB | drought, control | 1.34 | 0.2369 | 462 | 0.1876 |
| **CR1** | **SE** | **drought, control** | **1.63** | **0.0473** | **120** | **0.0758** |
| **CR1** | **SC** | **drought, control** | **2.53** | **0.0021** | **8183** | **0.0129** |

Table S3: Results of pairwise PERMANOVA on Euclidean distances comparing levels of the factor genotype within levels of the factors drought-treatment and root node. Significant differences between levels are shown in bold (P(perm) ≤ 0.05). In case of low numbers of possible unique permutations, we consider the P-values generated by Monte-Carlo testing (P(MC) ≤ 0.05) more meaningful here. The following variables were included: root surface area, stele surface area, cortex surface area, cortex:stele-ratio, cortical cell size, cortical file number, metaxylem number, total metaxylem surface area, aerenchyma surface area, and aerenchyma percent.

| Node order | Treatment | Genotype | t | P(perm) | perms | P(MC) |
| --- | --- | --- | --- | --- | --- | --- |
| CR1 | drought | WE, GB | 1.82 | 0.0977 | 10 | 0.0881 |
| CR1 | drought | WE, SE | 1.42 | 0.3013 | 10 | 0.1958 |
| CR1 | drought | WE, SC | 2.22 | 0.0732 | 15 | 0.0503 |
| CR1 | drought | GB, SE | 1.60 | 0.0991 | 10 | 0.1281 |
| **CR1** | **drought** | **GB, SC** | **2.05** | **0.0297** | **35** | **0.0504** |
| **CR1** | **drought** | **SE, SC** | **2.29** | **0.0255** | **35** | **0.0230** |
| CR1 | control | WE, GB | 2.06 | 0.1053 | 7 | 0.0440 |
| CR1 | control | WE, SE | 2.34 | 0.1042 | 10 | 0.0211 |
| CR1 | control | WE, SC | 2.46 | 0.1005 | 10 | 0.0206 |
| CR1 | control | GB, SE | 2.12 | 0.0990 | 7 | 0.0399 |
| CR1 | control | GB, SC | 1.32 | 0.1986 | 7 | 0.1964 |
| CR1 | control | SE, SC | 2.18 | 0.1014 | 10 | 0.0397 |
| CR2 | drought | WE, GB | 3.39 | 0.3258 | 3 | 0.0411 |
| CR2 | drought | WE, SE | 1.13 | 0.3321 | 3 | 0.3692 |
| **CR2** | **drought** | **WE, SC** | **2.89** | **0.0340** | **28** | **0.0049** |
| CR2 | drought | GB, SE | 1.89 | 0.3364 | 3 | 0.1244 |
| CR2 | drought | GB, SC | 1.54 | 0.0720 | 28 | 0.1307 |
| **CR2** | **drought** | **SE, SC** | **2.91** | **0.0358** | **28** | **0.0049** |
| **CR2** | **control** | **WE, GB** | **2.24** | **0.0165** | **680** | **0.0175** |
| CR2 | control | WE, SE | 1.66 | 0.2117 | 10 | 0.1447 |
| CR2 | control | WE, SC | 2.19 | 0.2018 | 10 | 0.0690 |
| **CR2** | **control** | **GB, SE** | **1.87** | **0.0360** | **120** | **0.0512** |
| CR2 | control | GB, SC | 1.18 | 0.2146 | 120 | 0.2597 |
| CR2 | control | SE, SC | 4.30 | 0.3305 | 3 | 0.0234 |
| CR3 | drought | WE, GB | 2.57 | 0.0667 | 15 | 0.0167 |
| CR3 | drought | WE, SE | 0.42 | 0.9779 | 45 | 0.8520 |
| CR3 | drought | WE, SC | 3.99 | 0.1019 | 10 | 0.0080 |
| CR3 | drought | GB, SE | 1.64 | 0.0845 | 495 | 0.0929 |
| **CR3** | **drought** | **GB, SC** | **4.39** | **0.0262** | **35** | **0.0016** |
| **CR3** | **drought** | **SE, SC** | **2.31** | **0.0104** | **165** | **0.0205** |
| **CR3** | **control** | **WE, GB** | **3.45** | **0.0007** | **4316** | **0.0007** |
| **CR3** | **control** | **WE, SE** | **3.12** | **0.0001** | **4320** | **0.0006** |
| CR3 | control | WE, SC | 1.29 | 0.1917 | 8360 | 0.1848 |
| **CR3** | **control** | **GB, SE** | **2.70** | **0.0002** | **8191** | **0.0008** |
| **CR3** | **control** | **GB, SC** | **1.94** | **0.0060** | **9839** | **0.0132** |
| **CR3** | **control** | **SE, SC** | **3.03** | **0.0002** | **9845** | **0.0001** |
| **CR2** | **drought** | **WE, GB** | **1.99** | **0.0075** | **126** | **0.0236** |
| **CR2** | **drought** | **WE, SE** | **2.24** | **0.0002** | **6713** | **0.0019** |
| **CR2** | **drought** | **WE, SC** | **2.49** | **0.0031** | **1987** | **0.0037** |
| CR2 | drought | GB, SE | 1.14 | 0.2808 | 2942 | 0.2835 |
| **CR2** | **drought** | **GB, SC** | **2.52** | **0.0037** | **715** | **0.0049** |
| **CR2** | **drought** | **SE, SC** | **3.96** | **0.0001** | **9882** | **0.0002** |
| **CR2** | **control** | **WE, GB** | **4.00** | **0.0055** | **210** | **0.0004** |
| CR2 | control | WE, SE | 2.59 | 0.0679 | 15 | 0.0215 |
| CR2 | control | WE, SC | 1.24 | 0.1997 | 3585 | 0.2231 |
| **CR2** | **control** | **GB, SE** | **3.91** | **0.0353** | **28** | **0.0010** |
| **CR2** | **control** | **GB, SC** | **3.88** | **0.0001** | **9094** | **0.0001** |
| CR2 | control | SE, SC | 1.12 | 0.2637 | 136 | 0.3017 |
| **CR1** | **drought** | **WE, GB** | **4.42** | **0.0107** | **84** | **0.0036** |
| CR1 | drought | WE, SE | 2.21 | 0.0970 | 10 | 0.0399 |
| **CR1** | **drought** | **WE, SC** | **4.64** | **0.0043** | **220** | **0.0004** |
| **CR1** | **drought** | **GB, SE** | **2.13** | **0.0111** | **84** | **0.0182** |
| **CR1** | **drought** | **GB, SC** | **7.54** | **0.0006** | **4322** | **0.0001** |
| **CR1** | **drought** | **SE, SC** | **5.37** | **0.0051** | **220** | **0.0001** |
| CR1 | control | WE, GB | 1.70 | 0.0624 | 4313 | 0.0808 |
| **CR1** | **control** | **WE, SE** | **1.88** | **0.0101** | **6626** | **0.0232** |
| **CR1** | **control** | **WE, SC** | **2.41** | **0.0011** | **8149** | **0.0105** |
| CR1 | control | GB, SE | 1.27 | 0.2433 | 1709 | 0.2138 |
| **CR1** | **control** | **GB, SC** | **1.74** | **0.0284** | **4311** | **0.0601** |
| **CR1** | **control** | **SE, SC** | **2.12** | **0.0051** | **6656** | **0.0233** |
